# Intestinal epithelial cell polarity defects in disease: lessons from microvillus inclusion disease

**DOI:** 10.1242/dmm.031088

**Published:** 2018-02-01

**Authors:** Kerstin Schneeberger, Sabrina Roth, Edward E. S. Nieuwenhuis, Sabine Middendorp

**Affiliations:** 1 Division of Paediatrics, Department of Paediatric Gastroenterology, Wilhelmina Children's Hospital, 3584 CT, Utrecht, The Netherlands; 2 Regenerative Medicine Center Utrecht, University Medical Centre (UMC) Utrecht, 3584 CT, Utrecht, The Netherlands

**Keywords:** Intestine, Polarity, Epithelial cells, Microvillus inclusion disease

## Abstract

The intestinal epithelium is a highly organized tissue. The establishment of epithelial cell polarity, with distinct apical and basolateral plasma membrane domains, is pivotal for both barrier formation and for the uptake and vectorial transport of nutrients. The establishment of cell polarity requires a specialized subcellular machinery to transport and recycle proteins to their appropriate location. In order to understand and treat polarity-associated diseases, it is necessary to understand epithelial cell-specific trafficking mechanisms. In this Review, we focus on cell polarity in the adult mammalian intestine. We discuss how intestinal epithelial polarity is established and maintained, and how disturbances in the trafficking machinery can lead to a polarity-associated disorder, microvillus inclusion disease (MVID). Furthermore, we discuss the recent developments in studying MVID, including the creation of genetically manipulated cell lines, mouse models and intestinal organoids, and their uses in basic and applied research.

## Abstract (precis)

**Summary:** Microvillus inclusion disease serves as a useful model to enhance our understanding of the intestinal trafficking and polarity machinery in health and disease.

## Introduction

The intestinal epithelium is a highly organized and rapidly self-renewing tissue with a proliferative crypt compartment and a differentiated villus compartment. The constant cellular turnover of the intestinal epithelium is maintained by stem cells, which reside at the bottom of the crypt and generate rapidly dividing daughter cells, the transit amplifying (TA) cells. The TA cells differentiate into various intestinal epithelial cell (IEC) types, such as Paneth cells, goblet cells, enteroendocrine cells, Tuft cells, M cells and the most prominent type, the absorptive enterocytes (Clevers, 2013).

Forming a continuous single-layered sheet, IECs separate the external environment from the internal one. The establishment of epithelial cell polarity with distinct apical and basolateral plasma membrane domains (described in detail below) is pivotal for barrier formation and for the uptake and vectorial transport (see Glossary, Box 1) of nutrients. The apical membrane of IECs faces the intestinal lumen, whereas the basolateral membrane borders neighboring cells and the underlying basement membrane, which covers the lamina propria (see Glossary, Box 1). Both membrane domains are composed of distinct proteins and lipids, which fulfill their distinct functions. These proteins and lipids are sorted and transported to the correct membrane domain via different intracellular routes, with cytoskeletal organization playing an important role in mediating this transport (Apodaca, 2001; Massey-Harroche, 2000).

**Box 1. Glossary**

**Adherens junctions:** or zona adherens; part of the junctional complex located in the lateral domain, consisting mostly of E-cadherin and mediating the strength of cell-cell adhesion.

**Apical recycling endosome (ARE):** a vesicle that contains apical membrane-destined cargo and RAB11A.

**Brush border:** densely packed microvilli on the apical side of enteroctyes.

**Caco-2 cells:** a cell line of heterogeneous human epithelial colorectal adenocarcinoma cells.

**Caco-2_BBE_:** a subclone of Caco-2 cells that uniformly expresses a highly ordered brush border (BB) cytoskeleton.

**Common recycling endosome (CRE):** a vesicle that contains apical or basolateral membrane-destined cargo and RAB11A.

**Desmosomal junctions:** part of the junctional complex, contains cadherin family members.

**Enteropathy:** any pathology of the intestine.

**Hemidesmosomes:** located at the basal part of the basolateral membrane, consist of dystroglycans and integrin receptors, facilitate the attachment to the basement membrane.

**Hypogammaglobulinemia:** a type of primary immunodeficiency resulting in severe reduction of gamma globulins in the blood due to a lack of antibody production.

**Junctional complex:** located in the lateral membrane just below the apical membrane, consists of tight, adherens and desmosomal junctions.

**Lamina propria:** connective tissue underlying the epithelium.

**Macropinocytosis:** a nonspecific cellular process to take in extracellular fluids by invagination of the plasma membrane.

**Microvilli:** extrusions from the apical membrane, supported by an actin filament network, to increase the absorptive surface area of cells.

**Microvillus inclusions:** intracellular vesicle-like structures that are internally (luminally) lined by microvilli, characteristic of microvillus inclusion disease.

**Paracellular transport:** the transfer of substances across an epithelium by passing through the intercellular space between the cells, controlled by junction complexes.

**Terminal web:** a filamentous structure composed primarily of actin filaments at the apical surface of epithelial cells that possess microvilli.

**Tight junctions:** or zona occludens; part of the junctional complex, located between the apical domain and the lateral surface domain, contains proteins of the claudin family and controls the paracellular transport of electrolytes and water.

**Total parenteral nutrition (TPN):** intravenous feeding that provides patients with all the fluid and the essential nutrients they need when feeding by mouth is inhibited.

**Vectorial transport:** transport of an ion or molecule across an epithelium in only one direction.

The mislocalization of epithelial proteins can disrupt the polarity and function of the epithelium and have far-reaching consequences for the health of cells and organisms. For example, the mislocalization of apical proteins in IECs leads to malnutrition, owing to the failure to properly absorb nutrients across the apical membrane, and to potentially fatal diarrheal disorders (Overeem et al., 2016). By contrast, the mislocalization of basolateral proteins correlates with loss of epithelial architecture, cancer development (Fatehullah et al., 2013), and with inflammatory bowel disease (Klunder et al., 2017).

In this Review, we focus on the structural regulation of polarity and intracellular transport mechanisms in IECs, the importance of which is illustrated by the pathophysiological defects in microvillus inclusion disease (MVID). MVID is a severe neonatal enteropathy (see Glossary, Box 1) that manifests mostly during the first days of life. It was first described in 1978 as a familial enteropathy characterized by protracted diarrhea from birth and failure to thrive (Davidson et al., 1978). Current treatment consists of life-long total parenteral nutrition (TPN; see Glossary, Box 1) and eventual small bowel and/or liver transplantation (Ruemmele et al., 2006). The pathological hallmarks of MVID are increased numbers of subapical vesicles, variable loss of microvilli (see Glossary, Box 1) and the presence of microvillus inclusions (see Glossary, Box 1) in villus enterocytes (Sherman et al., 2004) (Box 2). MVID is caused by heterogenous mutations in myosin Vb (*MYO5B*) (Müller et al., 2008; van der Velde et al., 2013) or syntaxin 3 (*STX3*) genes (Wiegerinck et al., 2014), which both encode proteins that function in the intracellular trafficking and membrane fusion cell machinery. Mutations in syntaxin binding protein 2 (*STXBP2*) also result in a MVID phenotype in the intestine (Stepensky et al., 2013; Vogel et al., 2017b) (Table 1).

**Box 2. Pathophysiology of microvillus inclusion disease**

The gold standard of MVID diagnosis is the morphological analysis of biopsies obtained from the small intestine of patients. On examination, MVID biopsies show villus atrophy with little crypt hyperplasia and the absence of strong inflammatory infiltrate in the lamina propria (Sherman et al., 2004). It has to be kept in mind that phenotypic changes can be intermingled with normal appearing epithelium.

**Histological characteristics of MVID**

• Absent or abnormal microvilli, which can be aberrant in form or reduced in number, in combination with discontinuity of the brush border and shortened, disoriented microvillus core rootlets

• Subapical staining for alkaline phosphatase (AP) (Lake, 1988)

• Periodic acid-Schiff (PAS) staining of apically located intracellular granules (Iancu et al., 2007)

• Subapical staining for CD10 (Koepsell and Talmon, 2010)

• Diffuse apical cytoplasmic staining for RAB11A in enterocytes (Talmon et al., 2012; Thoeni et al., 2013)

**Ultrastructural characteristics by electron microscopy (Iancu et al., 2007)**

• Subapical microvillus inclusions at the tip of the villi

• Diffuse microvillus atrophy and dystrophy

• Lateral membrane microvilli that project into the intercellular cleft

• Rod-like immature microvilli that reside intracellularly beneath the terminal web

• Abnormal vesicle-like organelles of various sizes, shapes and electron density, preferably within areas of microvilli-denuded apical surfaces

• Cytoplasmic inclusions lined by inward-pointing microvilli palisades, which feature irregular or rudimentary microvilli, and contain debris or amorphous components

• Lysosomes with heterogeneous contents. The number of lysosomes present correlates with the degree of membrane damage, suggesting they originate from autophagocytosis

**Extra-intestinal ultrastructural characterization**

Besides the intestine, microvillus inclusions can be identified in epithelial cells that line the stomach, gallbladder (Rhoads et al., 1991) and renal tubules (Cutz et al., 1997).

**Table 1.**
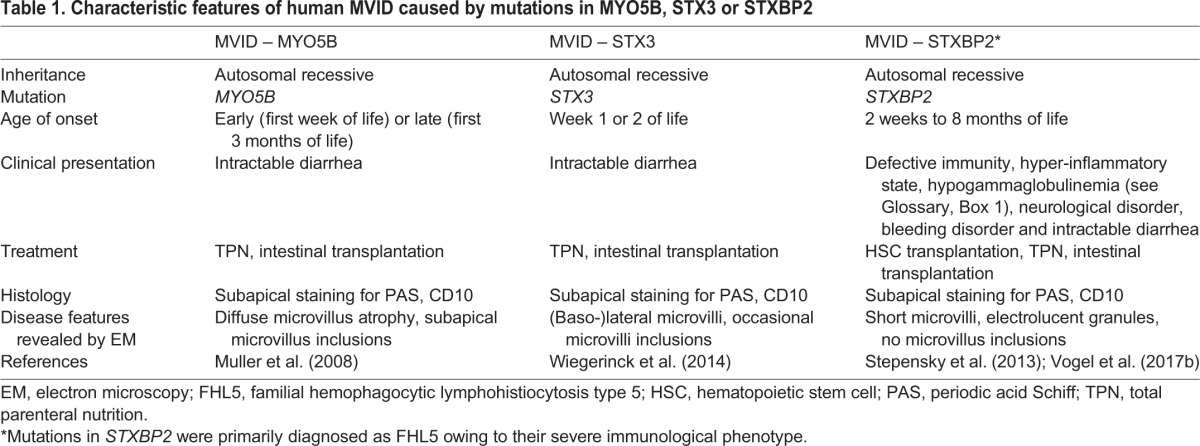
**Characteristic features of human MVID caused by mutations in MYO5B, STX3 or STXBP2**

The pathophysiological mechanism of MVID is currently not fully understood. Here, we review the recent developments in studying the pathophysiology of MVID using various approaches, including genetically manipulated cell lines and mouse models. We also propose a novel model to explain the pathological hallmarks of this disease, and discuss how recent insights from experimental models support it.

## Intestinal epithelial cell polarity

The intestine is lined by a simple columnar epithelium of polarized cells. The apical and basolateral membranes of all IECs are two biochemically and functionally distinct domains that each contain different protein and lipid compositions, which mediate their specialized functions. In this Review, we will specifically focus on enterocytes, the absorptive cells in the intestine, because the apical surface area of enterocytes is extended considerably by the formation of microvilli. The microvilli form a brush border (see Glossary, Box 1), which contains various apical proteins necessary for nutrient absorption and digestion. The establishment and maintenance of these distinct apical and basolateral membrane domains requires a highly specialized subcellular machinery, which traffics and recycles proteins to their appropriate location.

MYO5B and STX3 are both important mediators of the trafficking and membrane fusion machinery that maintains epithelial polarity, and defects in either of these proteins can cause MVID. As such, we use MVID as a model system to discuss the properties of the apical and basolateral membranes, and to examine how vectorial transport, membrane recycling and membrane fusion are regulated to maintain epithelial polarity.

### The apical membrane

The apical plasma membrane of IECs faces the intestinal lumen and has two main functions: the formation of a defensive barrier against pathogens, and the processing and uptake of nutrients. It consists of two layers, the inner one being rich in phosphatidylinositol-4,5-bisphosphate, and the outer one containing glycosphingolipids and cholesterol. The outer layer is able to form microdomains, also known as lipid rafts (Danielsen and Hansen, 2008). Proteins that have to be transported to the apical plasma membrane are modified post-translationally to feature apical sorting signals, such as *N*- and *O*-linked glycans and glycosylphosphatidylinositol anchors, and are then sorted and packaged into distinct transport carriers that leave the *trans-*Golgi network (TGN) (Rodriguez-Boulan et al., 2005). The way in which proteins are modified determines the route they take to the apical membrane; for example, via the lipid raft-dependent or -independent trafficking route (Jacob and Naim, 2001). Mutations interfering with *O*-linked glycosylation of the sucrase-isomaltase (SI) enzyme are believed to be associated with aberrant sorting of SI to the basolateral membrane in congenital sucrase-isomaltase deficiency (CSID) (Keiser et al., 2006).

As mentioned above, the microvilli extrude from the apical membrane of intestinal enterocytes. Each mature enterocyte contains ∼1000 microvilli, which are densely packed and together form the brush border (Mooseker, 1985). The brush border significantly increases the apical surface area, thereby facilitating efficient nutrient absorption and host defense against the luminal microbiota (Maroux et al., 1988; Mukherjee et al., 2008; Shifrin and Tyska, 2012).

The core of each microvillus consists of bundles of actin filaments that are interconnected by various proteins, including villin, plastin (fimbrin), espin and epidermal growth factor receptor kinase substrate 8 (ESP8) (Crawley et al., 2014; Heintzelman and Mooseker, 1992; Revenu et al., 2012). The microvilli are anchored to the subapical terminal web (see Glossary, Box 1) by myosin II (Heintzelman and Mooseker, 1992). Strikingly, several cytoskeletal genes, including those encoding villin, plastin and actin, are temporarily upregulated during enterocyte differentiation (Chang et al., 2008), and mouse models lacking myosin IA, myosin VI, ESP8, ezrin, espin, villin and/or plastin display defects in brush border formation (Casaletto et al., 2011; Ferrary et al., 1999; Grimm-Gunter et al., 2009; Hegan et al., 2012; Revenu et al., 2012; Saotome et al., 2004; Tocchetti et al., 2010; Tyska et al., 2005). The adhesion of the apical plasma membrane to the actin bundles in the core of the microvilli is established by myosin IA (Mazerik and Tyska, 2012), myosin VI (Hegan et al., 2012), and by members of the ezrin-radixin-moesin (ERM) family (Fehon et al., 2010).

In IECs, activated (phosphorylated) ezrin connects the plasma membrane to the actin bundles inside the microvilli (Bretscher et al., 2002, 1997; Smith et al., 2003). Several kinases have been implicated in the direct phosphorylation of ezrin at T567 in IECs, including protein kinase B2/Akt2, atypical protein kinase C-iota (aPKCι) (Wald et al., 2008), mammalian Sterile 20 (Ste20)-like kinase-4 (MST4; STK26) (Gloerich et al., 2012; ten Klooster et al., 2009), lymphocyte-oriented kinase and Ste20-like kinase (Viswanatha et al., 2012), all of which have been shown to be important for microvillus formation at the apical membrane. The polarity of the apical membrane of IECs also relies on the association with the CDC42/PAR complex, which is composed of the PAR6B (PARD6B), aPKCι, PAR3 (PARD3) and CDC42 proteins. The apical localization of the CDC42/PAR complex and the ezrin kinases was shown to be dependent on RAB11A and MYO5B (Dhekne et al., 2014; Michaux et al., 2016). In concordance, it was found that the polarity determinants CDC42, PAR6B and aPKCι, and the structural proteins ezrin and phospho-ezrin, were lost from the apical membrane and accumulated either in the cytoplasm or on the basal side of enterocytes in MVID patients, which suggests an inversion of cell polarity (Michaux et al., 2016).

### The basolateral membrane

The basolateral plasma membrane of IECs is rich in phosphatidylinositol-3,4,5-trisphosphate and is crucial for interactions between adjacent cells and with the basement membrane. Proteins that have to be transported to the basolateral membrane feature basolateral sorting signals, which are distinct from the apical sorting signals and mostly consist of simple peptide motifs located in the cytoplasmic domain of a protein (Weisz and Rodriguez-Boulan, 2009).

The lateral part of the basolateral membrane contains junctional complexes that tightly adhere adjacent cells to each other and control the paracellular transport (see Glossary, Box 1) in epithelia (Laukoetter et al., 2006). The junctional complexes (see Glossary, Box 1) consist of three components – tight junctions, adherens junctions and the desmosomal junctions (Farquhar and Palade, 1963; Giepmans and van Ijzendoorn, 2009; Shen et al., 2011) – whereas the basal part of the basolateral membrane contains hemidesmosomes (Stutzmann et al., 2000).

Although it is not yet clear whether basolateral membranes are affected in MVID, the mislocalization of basolateral proteins, such as transferrin receptor and α2-integrin to the cytoplasm has been reported in some MVID patients and mouse models (Schneeberger et al., 2015; Thoeni et al., 2013). In addition, microvilli have been found to be mislocalized to the basolateral membrane, particularly in patients with MVID caused by *STX3* mutations (Wiegerinck et al., 2014), and in two MVID mouse models, the enterocyte-specific *Rab8a*; *Rab11a* double, and the *Rab11a* single, knockout mice (Feng et al., 2017). These and other MVID mouse models are discussed later in this Review.

### Vectorial transport and membrane recycling

**Fig. 1.**
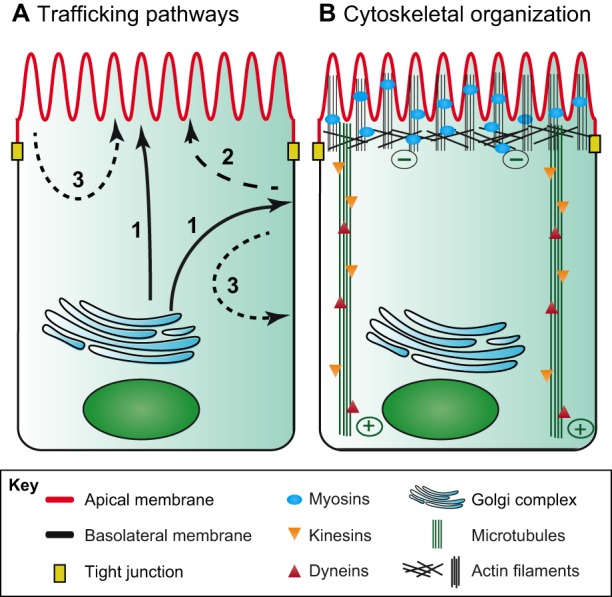
**Schematic overview of the intestinal trafficking machinery.** Schematics of polarized mouse enterocytes showing their cell features, cytoskeletal organization and trafficking routes. The apical surface is uppermost. (A) Apically and basolaterally destined proteins follow different pathways (denoted by arrows) to reach their target membrane. The biosynthetic route (route 1) is indicated in black line, the transcytotic route (route 2) in dashed line, and the recycling pathway (route 3) in dotted line. (B) Vesicle transport is mediated by the cytoskeleton. Long-distance transport occurs along microtubules, and is mediated by kinesin and dynein motor proteins. Short-distance transport occurs along actin filaments of the terminal web and is mediated by motor proteins of the myosin family.

During vectorial transport, cargo must pass through multiple compartments on its way towards the cell surface. These events are regulated by Rab proteins (44 subfamilies in humans), which modulate cargo selection and the tethering and fusion of vesicles with their target membranes (Apodaca, 2001). The establishment and maintenance of these distinct apical and basolateral membrane domains requires a highly specialized subcellular machinery that ensures that proteins are transported and recycled to their appropriate location. Apical proteins use a direct (biosynthetic) or indirect (transcytotic) route to reach their target membrane, whereas basolateral proteins use only the direct pathway (Le Bivic et al., 1990; Matter et al., 1990). Additionally, proteins from both plasma membrane domains can be endocytosed and transported back to their respective membranes via the recycling pathway (Golachowska et al., 2010; Utech et al., 2010) (Fig. 1A).

In the direct (biosynthetic) route (Fig. 1A, pathway 1), proteins that are synthesized in the endoplasmic reticulum (ER) are transferred via the Golgi complex to the *trans*-Golgi network. Here, proteins are sorted into distinct apical and basolateral endosomal carrier vesicles and are then transported to either the apical or the basolateral plasma membrane (Massey-Harroche, 2000). Apically targeted proteins passage through the common recycling endosome (CRE; see Glossary, Box 1) or through the apical recycling endosome (ARE; see Glossary, Box 1). Basolateral targeted proteins traffic through the CRE before reaching the basolateral plasma membrane (Le Bivic et al., 1990; Matter et al., 1990).

In the indirect (transcytotic) pathway (Fig. 1A, pathway 2), newly synthesized apically destined proteins are delivered first to the basolateral plasma membrane. After a variable time on the basolateral membrane, they are endocytosed and delivered to their apical target membrane by transcytosis. Again, this could happen by passaging though endosomal compartments such as the CRE and ARE (Matter et al., 1990).

In the recycling pathway (Fig. 1A, pathway 3), membrane proteins are endocytosed into apical early endosomes or into basolateral early endosomes (Fujita et al., 1990). Subsequently, three different fates are possible. First, the proteins can be targeted for degradation, whereby the endosomal cargo is transferred to lysosomes, which fuse to form late endosomes (Antileo et al., 2013). Second, endocytosed proteins can be rapidly recycled back to their respective membranes, which generally happens via RAB4-positive endosomal compartments (Rapetti-Mauss et al., 2013; Somsel Rodman and Wandinger-Ness, 2000). Third, endocytosed proteins can undergo slow recycling back to their respective membranes. In this route, apical and basolateral endocytosed cargo is first redirected to the CRE, where it is sorted again. Proteins are then directed to distinct apical and basolateral trafficking pathways, and apical proteins can passage through the ARE, which acts as an intermediate compartment (Golachowska et al., 2010; Rapetti-Mauss et al., 2013).

Experimental findings have identified several proteins that are associated with apical endosomal trafficking and their roles in IEC polarity. Myosin Vb (MYO5B), a motor protein crucial for endosomal transport along the cytoskeleton (discussed further below), binds to both RAB11A and RAB8A in a yeast two-hybrid screen, and colocalizes with these RAB proteins in polarized MDCK and HeLa cells (Hales et al., 2002; Lapierre et al., 2001; Roland et al., 2007). In *in vitro* studies using polarized IECs, MYO5B, RAB11A and RAB8A have been reported to associate with AREs, where they control the activity of CDC42 (Bryant et al., 2010). However, in enterocytes from MVID patients and in MYO5B-mutated Caco-2 cells (see Glossary, Box 1), RAB11A-positive AREs are mislocalized (Dhekne et al., 2014; Szperl et al., 2011). Additional studies have used MYO5B mutant proteins that are unable to bind to either RAB8A or RAB11A, which result in distinct microvillus structural defects (Knowles et al., 2014; Vogel et al., 2015), indicating that RAB11A- and RAB8A-positive AREs play a pivotal role in IEC polarity. Polarized Caco2-_BBE_ cells (see Glossary, Box 1) showed a loss of microvilli upon knockdown of MYO5B. Re-expression of a specific MYO5B mutant that cannot bind to RAB11A, rescued the loss of microvilli, although it caused the formation of microvillus inclusions. By contrast, re-expression of a RAB8A binding-deficient MYO5B mutant only partly rescued microvilli loss, and no inclusions were observed in the cells. Together, these data show that MYO5B-RAB8A binding is important for microvilli formation, and that the disruption of the MYO5B-RAB11A interaction is responsible for the formation of microvillus inclusions (Knowles et al., 2014).

### Cytoskeletal organization

In all trafficking routes, the transport of the distinct apical and basolateral carrier vesicles depends on the cytoskeleton and occurs along microtubules and actin filaments (Gilbert et al., 1991; Rodriguez-Boulan et al., 2005) (Fig. 1B). Microtubules run through the cytoplasm of the cells from the apical to the basal side, and interact with actin filaments at the periphery (Gilbert et al., 1991; Sandoz et al., 1986). The minus ends of microtubules face the apex of the cell, and the plus ends the basal side near the Golgi complex (Akhmanova and Hoogenraad, 2015; Dammermann et al., 2003). Long-distance transport along microtubules is mainly driven by two types of motor proteins: dyneins, which are minus end directed, and kinesin family proteins, which are mainly plus end directed (Brown, 1999; Gilbert et al., 1991; McNiven and Marlowe, 1999) (Fig. 1B). Recently, it has been shown that the organization of the apico-basal microtubules in polarized IECs is regulated by the direct binding of calmodulin-regulated spectrin-associated protein 3 (CAMSAP3) to the spectraplankin protein actin cross-linking factor 7 (ACF7) (Noordstra et al., 2016; Toya et al., 2016). Experiments in Caco-2 cells have demonstrated that CAMSAP3 strongly localizes to the apical cell membrane and tethers the minus ends of microtubules to the apical side (Toya et al., 2016). Loss of CAMSAP3 leads to RAB11A mislocalization and to the inhibition of brush border formation, whereas loss of ACF7 affects the lumen formation in three-dimensionally cultured Caco-2 cells (Noordstra et al., 2016). Additionally, the disassembly of microtubules by colchicine treatment leads to polarity defects in IECs *in vivo* and *in vitro* (Achler et al., 1989; Gilbert et al., 1991). Together, these findings confirm the importance of microtubule organization for proper IEC polarity.

The actin cytoskeleton is made up of shorter filaments that form a dense network located underneath the plasma membranes. Short-distance transport along actin filaments is mediated by myosin motor proteins (McNiven and Marlowe, 1999) (Fig. 1B). Myosin VI is the only myosin motor that moves towards the minus ends of actin filaments, which are anchored in the subapical terminal web. Myosin VI mediates clathrin-dependent endocytosis of several apical proteins and trafficking to the subapical endosome (Ameen and Apodaca, 2007; Hegan et al., 2012). Myosin V (MYO5) is present in the terminal web and at the distal ends of the microvilli (Heintzelman et al., 1994) and has three subclasses (MYO5A, MYO5B and MYO5C). MYO5B plays a particularly important role in the intestinal epithelium. It works as a tether, mediating the transport of endosomes toward the plus ends of actin filaments in the tip of the microvilli, and thereby has a crucial role in the establishment and maintenance of IEC polarity (Kapitein et al., 2013; Roland et al., 2011).

### Membrane fusion

**Fig. 2.**
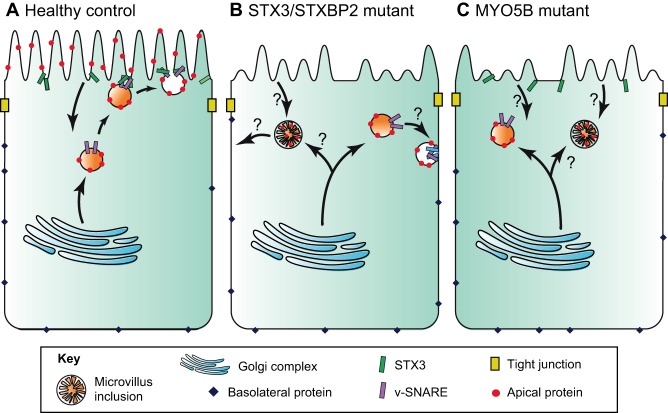
**Model to explain MVID pathology caused by mutations in *STX3*, *STXBP2* or *MYO5B*.** The panels depict healthy control and mutant human enterocytes, showing endosomal trafficking routes. The apical surface is uppermost. (A) In healthy (control) enterocytes, vesicles containing apical proteins travel from the Golgi complex to the apical membrane. These vesicles fuse with the apical membrane through the interaction of a v-SNARE with the t-SNARE, syntaxin 3 (STX3) and its binding partner STXBP2. (B) STX3/STXBP2-deficient enterocytes fail to deliver apically destined vesicles to the apical membrane and might instead deliver apical recycling endosomes (AREs) that contain apical proteins to the basolateral membrane, leading to the formation of basolateral microvilli. In the apical membrane, microvilli are distorted or absent and are instead accumulating in microvillus inclusions, which are formed by a yet unresolved mechanism. (C) *MYO5B* mutant enterocytes also fail to deliver apically destined vesicles to the apical membrane, lack apical microvilli and are prone to form microvillus inclusions. Question marks in B and C indicate unresolved mechanisms.

Once the AREs are in close proximity to the apical membrane; following their transport along the microtubule and actin cytoskeleton, their protein contents have to be released or incorporated into the membrane. This process involves the AREs docking to, and subsequently fusing with, the target membrane, which is mediated by soluble N-ethylmaleimide-sensitive factor attachment receptor (SNARE) complexes (Hong and Lev, 2014). SNARE complexes are composed of vesicle-SNAREs (v-SNAREs), which are associated with the cargo-loaded vesicle, and target-SNAREs (t-SNAREs), which are transmembrane proteins in the plasma membrane. T-SNAREs consist of syntaxins and synaptosomal-associated proteins (SNAP), whereas v-SNAREs are a family of vesicle-associated membrane proteins (VAMPs). In IECs, vesicle tethering and fusion is controlled by the v-SNAREs synaptotagmin-like protein 4a (SLP4A) and vesicle-associated membrane protein 7 (VAMP7), in conjunction with RAB27A/RAB3/RAB8A/RAB11A proteins and the t-SNARE syntaxin-3 (STX3) (Breuza et al., 2000; Delgrossi et al., 1997; Galli et al., 1998; Pocard et al., 2007; Riento et al., 1998; Vogel et al., 2015; Wiegerinck et al., 2014). Furthermore, the Sec1-related protein syntaxin binding protein 2 (STXBP2), also known as Sec1/Munc18-like protein (MUNC18-2), mediates binding of SLP4A with STX3 at the apical plasma membrane of IECs, where it regulates the accessibility of its SNARE partners (Riento et al., 1998; Vogel et al., 2017b). It has been recently shown that the apical exocytosis route requires MYO5B, RAB11A, RAB8A, SLP4A, VAMP7, STXBP2 and STX3, which together regulate the recycling and localization of several apical membrane proteins, such as sodium-hydrogen exchanger 3 (NHE3), cystic fibrosis transmembrane conductance regulator (CFTR) and solute carrier family 2 member 5 (GLUT5) (Vogel et al., 2015). Mutations in *STX3* or *STXBP2* cause MVID and result in severe diarrhea (Stepensky et al., 2013; Vogel et al., 2017b; Wiegerinck et al., 2014), indicating that, in addition to MYO5B-regulated trafficking, the membrane fusion machinery also has an important role in the maintenance of IEC polarity (Fig. 2).

## Disrupted intestinal epithelial cell polarity in MVID

As mentioned above, most aspects of IEC polarity are affected in MVID. Thus, we use MVID as a model to understand how IEC polarity is regulated. Currently, the pathological hallmarks of MVID are explained by three distinct models: the trafficking, the recycling and the local induction models. In addition to these, we propose a novel mechanistic model that combines elements from these three models to explain all the hallmarks of MVID. In this section, we discuss the characteristics and pathophysiology of MVID and the experimental model systems that are being used to study MVID, including genetically modified cell lines and mouse models.

### Characteristics of MVID

MVID was previously known as Davidson's disease, congenital microvillus atrophy, and as intestinal microvillus dystrophy (Cutz et al., 1989; Phillips and Schmitz, 1992). MVID clusters in certain ethnic groups, including Arabs (Phillips and Schmitz, 1992), Iraqi Jews (Straussberg et al., 1997) and the American Navajo (Pohl et al., 1999), owing to consanguinity or a small gene pool. Based on the time of onset, two distinct forms of MVID can be distinguished: early-onset MVID, which occurs within the first week of life, and late-onset MVID, which occurs within the first 3 months of life (Müller et al., 2008; Phillips and Schmitz, 1992; Raafat et al., 1994). Affected newborns present with extremely watery diarrhea, dehydration and weight loss (Rhoads et al., 1991; Shahid et al., 2012).

MVID belongs to the congenital diarrheal disorders (CDD), which are subdivided into secretory, osmotic and mixed-type diarrhea, and can be either of epithelial or of immunological origin (Canani et al., 2015). Examples of CDD include epithelial dysplasia (tufting enteropathy), chloride or sodium diarrhea, Na-H-exchange deficiency, glucose-galactose malabsorption and SI deficiency (Overeem et al., 2016; Ruemmele et al., 2006).

It has been shown that MVID can be caused by mutations in either *MYO5B* (which occur in ∼90% of MVID patients) (Müller et al., 2008; van der Velde et al., 2013) or in *STX3* (two patients described so far) (Wiegerinck et al., 2014). Both genes encode proteins that function in the intracellular trafficking machinery of epithelial cells in general (Fig. 2). However, the early and most severe clinical manifestations of MVID are mainly restricted to the intestinal epithelium. In addition, patients with mutations in *STXBP2* also have MVID in addition to their main clinical manifestation of familial hemophagocytic lymphohistiocytosis type 5 (FHL5) (Stepensky et al., 2013; Vogel et al., 2017b). Although many variations of MVID pathology have been described, the most common clinical and histological hallmarks of the disease are summarized in Box 2 and Table 1.

Intestinal biopsies of MVID patients exhibit villus atrophy, microvillus atrophy and the redistribution of CD10 and periodic acid Schiff (PAS)-stained material from the brush border to intracellular sites in the enterocytes (Phillips et al., 2000). In addition to CD10 and PAS staining, it has been proposed that the subapical localization of villin (Shillingford et al., 2015) or RAB11 (Talmon et al., 2012) could be used as markers for MVID diagnosis. Analysis by electron microscopy (EM) is needed to reveal microvillus inclusions in the cytoplasm of enterocytes. However, the frequency of such inclusions can be very low and they are mainly restricted to the tips of the villi. For example, the enterocytes of some MVID patients exhibit features of the disease that are visible by light microscopy, such as subapical PAS and CD10 staining, but do not show microvillus inclusions under EM (Mierau et al., 2001). This variability might reflect the limitations of detection by EM or might be due to the heterogeneity of the disease, potentially caused by the variability of mutations in *MYO5B* (Szperl et al., 2011), *STX3* (Wiegerinck et al., 2014) and *STXBP2* (Stepensky et al., 2013; Vogel et al., 2017b).

One might argue that much of the MVID phenotype results from the loss of microvilli and to the consequent loss of enterocyte surface area for efficient nutrient absorption. However, surface area reduction in MVID patients does not fully explain the MVID phenotype, and some patients even show normal-appearing microvilli (Vogel et al., 2017a). The intestines of most MVID patients are in a secretory state, and excrete electrolytes and water even without enteral feeding (Davidson et al., 1978). The mislocalization of apical membrane proteins required for nutrient digestion, absorption and electrolyte transport might further explain their clinical symptoms (Ameen and Salas, 2000; Dhekne et al., 2014; Kravtsov et al., 2014; Michail et al., 1998; Müller et al., 2008; Vogel et al., 2017a).

A 16-fold expansion of the vesicular compartment, containing electron-dense vesicles and displaced mitochondria, has also been observed in intestinal biopsies from MVID patients (Ameen and Salas, 2000). Additional studies of MVID patient biopsies have revealed the aberrant localization of the apical proteins SI, sodium-hydrogen exchanger 2 (NHE2), NHE3, alkaline phosphatase (ALP), CFTR, dipeptidyl peptidase IV (DPP-IV), sodium-glucose linked transporter 1 (SGLT1) and phosphoinositide-dependent protein kinase 1 (PDK1) (Ameen and Salas, 2000; Dhekne et al., 2014; Kravtsov et al., 2014; Michail et al., 1998; Müller et al., 2008; Vogel et al., 2017a). Nevertheless, there is *in vitro* evidence to suggest that DPP-IV, SI and ALP are apically localized via a MYO5B/STX3-independent route, as these proteins are not mislocalized in Caco-2 cells that lack functioning MYO5B or STX3 proteins (Vogel et al., 2017a, 2015). In addition, in the gut biopsies of some MVID patients, the basolateral membrane protein Na/K-ATPase was localized in the same pattern as it was in the gut biopsies of healthy controls (Ameen and Salas, 2000). The discrepancy between the results of these studies is most likely caused by the enormous variability of apical membrane morphology in MVID, which can range from membrane being devoid of microvilli and harboring numerous microvillus inclusion bodies to areas of membrane that appear to be morphologically healthy (Phillips and Schmitz, 1992; Vogel et al., 2017a). Furthermore, it remains to be elucidated whether the mislocalization of membrane proteins is a consequence of microvilli loss or of mutant MYO5B inappropriately altering the localization of these proteins.

### Mechanistic models for MVID

**Fig. 3.**
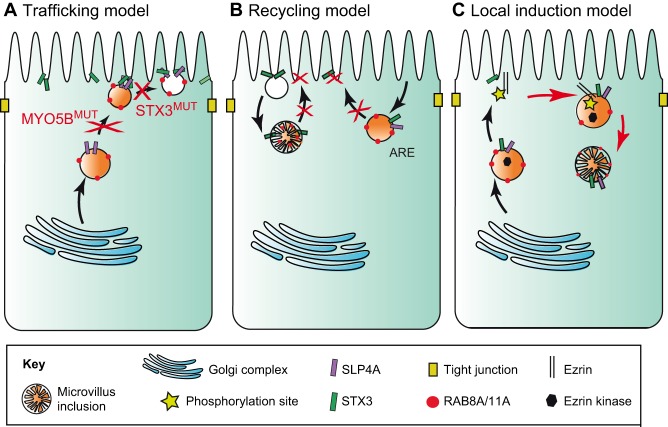
**Three models to explain the pathological hallmarks of MVID.** The panels depict human enterocytes, showing endosomal trafficking routes (black arrows). The apical surface is uppermost. (A) In the trafficking model, defects (depicted by red crosses) in vesicle trafficking (caused by *MYO5B* mutations, MYO5B^MUT^) or delivery (caused by *STX3* mutations, STX3^MUT^) result in the subapical accumulation of vesicles and in the lack of appropriately polarized apical proteins. (B) In the recycling model, defects in the recycling and delivery of apical recycling endosomes (AREs) result in the subapical accumulation of apical proteins and in the formation of microvilli-containing macropinosomes. (C) In the local induction model, MVID results in colocalization of ezrin and ezrin kinases in the subapically accumulated AREs to create a signaling platform that results in local ectopic microvillus formation, which leads to the formation of microvillus inclusions (red arrows). In healthy cells, ezrin kinases are transported to the apical membrane where they activate ezrin by phosphorylation to induce microvillus formation.

Taking the results from morphological and genetic studies together, three mechanistic models have been proposed to explain the main pathological hallmarks of MVID: lack of microvilli, protein mislocalization and the formation of microvillus inclusions (Fig. 3). The three models have been based on several studies using cell lines and MVID mouse models. We briefly explain each model below and propose a new combined model to explain the pathology of MVID.

#### Trafficking model

In this model, apical trafficking pathways are MYO5B- and STX3-dependent, and mutations lead to a failure of vesicle trafficking or to a failure of the fusion of vesicles with the plasma membrane (Ameen and Salas, 2000; Roland et al., 2011; Wiegerinck et al., 2014). This failure of vesicle trafficking causes vesicles that contain apical proteins to accumulate subapically (Fig. 3A).

#### Recycling model

In the recycling model, endocytosis and the subsequent recycling of apical proteins results in the internalization of apical proteins. In MVID, the re-expression of recycled apical proteins is compromised by the lack of vesicle trafficking or fusion by dysfunctional MYO5B or STX3. This model can also involve macropinocytosis (see Glossary, Box 1), which can lead to the engulfment of large stretches of the plasma membrane, resulting in intracellular macropinosomes, potentially lined by microvilli (Davidson et al., 1978; Knowles et al., 2014; Reinshagen et al., 2002; Roland et al., 2011) (Fig. 3B). It is currently not resolved whether microvillus inclusions in the enterocytes of MVID patients are in fact macropinosomes or if they are formed via a different mechanism.

#### Local induction model

As previously discussed, MYO5B is required for the localization of RAB11A-positive AREs, which contain various signaling molecules, such as PDK1, PKCi and MST4 (Dhekne et al., 2014; Kravtsov et al., 2014; Szperl et al., 2011). Inside the AREs, these kinases colocalize with ezrin. This local induction model proposes that in MVID, RAB11A-positive AREs accumulate and function as a subapical signaling platform to induce ectopic intracellular microvillus formation (Cutz et al., 1989; Dhekne et al., 2014; Vogel et al., 2015) (Fig. 3C).

#### The combined model

Here, we propose a novel hybrid model that combines all three models above. Vesicles, either derived from the Golgi complex or from apical/basolateral membranes by endocytosis, are transported from the perinuclear region to the cell periphery via microtubules and actin filaments. MYO5B is required to tether the vesicles to the actin filaments in the subapical area. At the plasma membrane, either docking or fusion of the vesicles via STX3 and STXBP2 is required to colocalize ezrin kinases in close proximity to ezrin. In MVID, where either MYO5B or STX3 function is disturbed, ARE cannot fuse with the apical membrane, which leads to the subapical accumulation of vesicles and ultimately gives rise to mislocalized microvilli and/or microvillus inclusions. Thus, microvillus inclusions are vesicles that have either sequestered the microvilli from the apical membrane by macropinocytosis or have formed ectopic microvilli owing to the colocalization of ezrin and ezrin kinases in the subapical compartment.

However, one issue that argues against the proposed combined model is that one would expect the microvilli to be internalized across the entire apical surface, which would result in the formation of numerous microvillus inclusions in each cell. By contrast, inclusions are mainly found at the villus tips and are rarely found in the intestinal crypts. Furthermore, inclusions are only observed in 10-20% of the enterocytes of *MYO5B* mutant MVID patients and of *Myo5b* knockout mice (Cutz et al., 1989; Schneeberger et al., 2015). It is possible that this phenotypic variation is caused by the initial, rapid degradation of inclusions in the lysosomes of immature IECs. When the degradation machinery is exhausted over time, inclusions start to accumulate and are therefore predominantly found in mature IECs at the tips of the villi. Because microvillus inclusions are not reliably present upon pathological examination of IECs from MVID patients (Mendes et al., 2014), additional genetic testing, such as of *MYO5B*, *STX3* and *STXBP2*, should be performed to confirm a MVID diagnosis. When these genes are not affected, a panel of genes involved in other CDDs (Canani et al., 2015; Overeem et al., 2016) or genome-wide sequencing should be considered.

### Investigating the polarity of intestinal epithelial cells

Over the past few years, several studies have added to our understanding of MVID pathophysiology (Kravtsov et al., 2014; Ruemmele et al., 2010; Thoeni et al., 2013; Vogel et al., 2017a, 2015). In addition to studying primary tissue obtained by biopsy from MVID patients, genetically modified cell lines and animal models have also been developed and used to study MVID pathology, which are summarized below.

#### Cell lines

The *in vitro* systems used to study intestinal polarity and trafficking mostly derive from human colorectal adenocarcinomas, such as Caco-2 and LS174 cells. Caco-2 and Caco-2_BBE_ cells spontaneously polarize *in vitro*, when cultured in a tight monolayer for over 2 weeks (Peterson et al., 1993; Peterson and Mooseker, 1992, 1993). They form a mature brush border, express small intestine-specific enzymes, such as SI, and use trafficking routes specific for polarized cells, including direct biosynthetic trafficking and transcytosis (Chantret et al., 1988; Fleet et al., 2003; Gilbert et al., 1991; Le Bivic et al., 1990; Matter et al., 1990). Several research groups have used Caco-2 or Caco-2_BBE_ cells to study the effect of *MYO5B* knockdown (KD) and/or re-expression of various *MYO5B* mutants. Most of the *MYO5B* KD models develop the main characteristics of MVID, i.e. loss of microvilli, mislocalization of apical and basolateral proteins, and microvillus inclusions (Kravtsov et al., 2014; Ruemmele et al., 2010; Thoeni et al., 2013; Vogel et al., 2017a, 2015).

In one study, MYO5B-depleted Caco-2 cells formed very few microvilli; however, microvillus inclusions were not observed (Knowles et al., 2014). This could be caused by the incomplete KD of *MYO5B*, as MYO5B protein levels decreased by ∼50% in this study, whereas in other studies that achieved complete *MYO5B* KD or genome-edited Caco-2 cells, microvillus inclusions formed in the *MYO5B*-deficient cells (Kravtsov et al., 2014; Thoeni et al., 2013; Vogel et al., 2017a). In addition, the knock-in of the specific *MYO5B* mutation 1125G>A, or overexpression of the *STX3* truncating mutations (AA1-125 and AA1-247), in Caco-2 cells all resulted in a MVID phenotype (Vogel et al., 2015; Wiegerinck et al., 2014). However, as it has been shown that ±1% of Caco-2 cells exhibit spontaneous microvillus inclusions, the use of this cell line makes it difficult to reach conclusions about the direct effects of the introduced mutations.

The LS174-W4 cell line is also suitable to study intestinal epithelial polarization. The parental LS174T cells derive from a human colon adenocarcinoma (Kahan et al., 1976), and the daughter cell line, LS174-W4, was manipulated to stably express LKB1 and to inducibly express the pseudokinase, strad-α, both of which are required for ezrin phosphorylation (ten Klooster et al., 2009). Treatment of LS174-W4 cells with doxycyclin induces strad-α expression, which results in the polarization of individual cells, including the formation of a brush border and distinct apical and basolateral membrane domains (Baas et al., 2004; ten Klooster et al., 2009). *MYO5B* KD in LS174-W4 cells resulted in the loss of microvilli and in mislocalized RAB11A-positive ARE-containing apical proteins (Dhekne et al., 2014).

In summary, *MYO5B* KD in LS174-W4 and in Caco-2/Caco-2_BBE_ cells recapitulates the polarity defects that are observed in the enterocytes of MVID patients, albeit to varying degrees (Dhekne et al., 2014; Knowles et al., 2014; Kravtsov and Ameen, 2013; Müller et al., 2008; Thoeni et al., 2013; Vogel et al., 2015).

#### Animal models

Over recent years, various mouse models, as well as a zebrafish model (Sidhaye et al., 2016), have been used to study MVID. Here, we focus on the mouse models of this disease and summarize the findings in Table 2. In earlier mouse models of MVID, which were generated by *Rab8a*, *Rab11a* and *Cdc42* mutations, or combinations thereof, the animals failed to thrive due to unresolved causes (Feng et al., 2017; Melendez et al., 2013; Sakamori et al., 2012; Sato et al., 2007; Sobajima et al., 2014; Yu et al., 2014). Microscopically, these MVID mouse models are characterized by abnormal microvilli, the mislocalization of apical proteins and by microvillus inclusions (Table 2). However, mutations in *RAB8A*, *RAB11A* and *CDC42* have, as yet, not been found in MVID patients. (Talmon et al., 2012; Thoeni et al., 2013; Vogel et al., 2017a). Most importantly, except for the *Rab8a*-deficient mice, none of these mouse models died from secretory diarrhea, which is the most devastating hallmark of human MVID (Table 2).

**Table 2.**
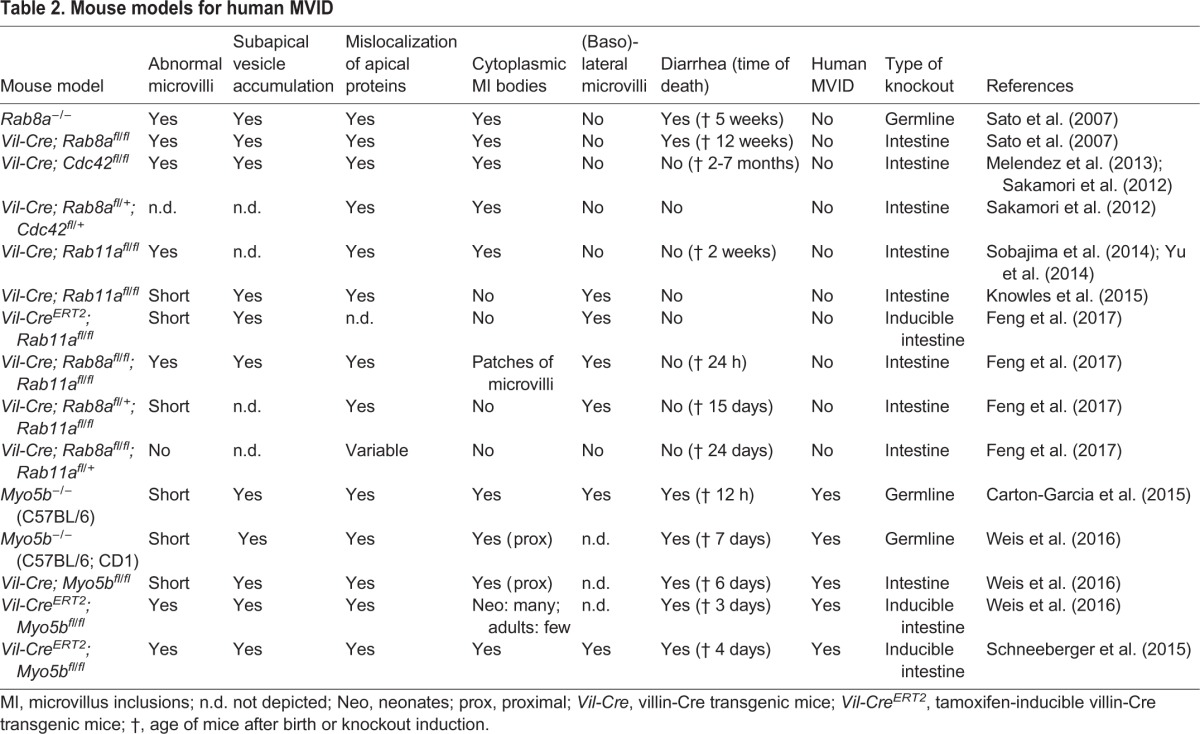
**Mouse models for human MVID**

Recently, three independent papers described five different *Myo5b*-deficient mouse models, which were generated by using either an intestine-specific inducible *Myo5b* knockout (Schneeberger et al., 2015; Weis et al., 2016) or a germline/constitutively targeted *Myo5b* knockout (Cartón-García et al., 2015; Weis et al., 2016). All five *Myo5b*-deficient models recapitulated the human MVID phenotype: atrophy, complete loss or fusion of microvilli, a varying degree of cytoplasmic microvillus inclusions, the mislocalization of apical proteins, and the subapical accumulation of vesicles in enterocytes. Most importantly, all *Myo5b*-deficient mice died shortly after birth or after mutation induction due to severe secretory diarrhea. These findings demonstrated, for the first time, that lack of MYO5B causes all hallmarks of human MVID, including secretory diarrhea (Cartón-García et al., 2015; Schneeberger et al., 2015; Weis et al., 2016). Interestingly, the induction of *Myo5b* deficiency in adult mice resulted in the presence of fewer microvillus inclusions in enterocytes compared to neonatally induced mice, in which many inclusions were observed. This indicates that the timing of mutation induction might have differential effects, although it is not yet understood how this is regulated (Weis et al., 2016). It might reflect the findings in *MYO5B* KD Caco-2_BBE_ cells and human MVID enterocytes that maturation of MYO5B-deficient cells is retarded (Kravtsov et al., 2016).

In addition, the cytoplasmic mislocalization of apical proteins, such as of phospho-ezrin, CD10 and actin, and the subapical accumulation of electron-dense and translucent vesicles were observed in enterocytes of all five *Myo5b*-deficient mouse models. Interestingly, the enterocytic basolateral localization of E-cadherin and of Na/K-ATPase was unaffected in the germline and intestine-specific *Myo5b* knockout mouse models (Cartón-García et al., 2015; Weis et al., 2016). However, both tamoxifen-inducible *Myo5b* knockout models displayed partly aberrant localization of E-cadherin and Na/K-ATPase in enterocytes (Schneeberger et al., 2015; Weis et al., 2016). The presence of microvilli at the lateral membrane was described in only one of the five *Myo5b* knockout mouse models (Cartón-García et al., 2015). These phenotypic differences between the five *Myo5b*-deficient mouse models are currently not yet understood, and might simply reflect the variation in genetic background, age or timing of induction.

More studies are needed to determine whether the age of onset (Weis et al., 2016), maturation stage of enterocytes (Kravtsov et al., 2016) and/or cephalocaudal location (Weis et al., 2016) is correlated to the severity of the disease phenotype. For example, the inducible *Myo5b-*deficient mouse models can be used to study the sequence of events of the various disease-specific hallmarks, such as the origin of microvillus inclusions and the onset of secretory diarrhea, in an age-, time- and location-specific manner.

In summary, these *Myo5b*-deficient mouse models can be used to further investigate the molecular mechanisms underlying MVID and represent unique tools for the development and testing of novel therapeutic approaches.

#### Organoids

The generation of a three-dimensional primary intestinal culture system to create intestinal organoids has created new and exciting opportunities for studying IEC polarity. Organoids can be grown from single intestinal murine or human epithelial stem cells (Sato et al., 2011, 2009). When grown in an extracellular matrix, such as matrigel, and supplemented with the right growth factors, intestinal stem cells grow to form a three-dimensional mini-intestine. This purely epithelial structure closely resembles the *in vivo* tissue (Middendorp et al., 2014; Sato et al., 2011, 2009), contains all of the main epithelial intestinal cell types and location-specific functional proteins and can be infinitely expanded without acquiring phenotypic or genetic abnormalities (Blokzijl et al., 2016; Middendorp et al., 2014). Enterocytes in these organoids form a mature brush border and express apical and basolateral proteins on their respective membranes (Sato et al., 2011, 2009). More recently, a new technique to culture organoids in a two-dimensional system has been established (Moon et al., 2014; VanDussen et al., 2014; Vogel et al., 2017b). In this system, cells form a monolayer with distinct apical and basolateral membrane domains, allowing both domains to be easily accessed and manipulated.

Organoids derived from patients with polarity-associated diseases, such as MVID, cystic fibrosis (CF) or multiple intestinal atresia with a combined immunodeficiency (MIA-CID), faithfully recapitulate the disease phenotype (Bigorgne et al., 2014; Dekkers et al., 2013; Vogel et al., 2017b; Wiegerinck et al., 2014). For example, intestinal organoids derived from MVID patients display microvillus atrophy, microvillus inclusions and subapical accumulations of vesicles. In addition, organoids derived from various MVID mouse models have been used to study the effects of the disease on stem cell proliferation and differentiation (Feng et al., 2017; Schneeberger et al., 2015). As such, organoids derived from MVID patients or from mouse models provide a promising new tool for basic research on intestinal epithelial trafficking and the polarity machinery in healthy tissue, and for studying polarity-associated diseases in a patient-specific manner. Furthermore, the genetic manipulation of organoids by the use of viral transduction, bacterial artificial chromosome (BAC) transfection or the CRISPR/Cas9 gene editing system (Koo et al., 2013, 2012; Schwank et al., 2013a,b) provide tools with which to knockdown, overexpress or even repair genes to study their functions, in a polarized epithelial system that closely resembles the *in vivo* organ.

## Conclusion

The plasma membrane of polarized intestinal epithelial cells contains distinct apical and basolateral domains with specialized functions. Much of our knowledge on how these distinct membrane domains are established and maintained has been gained from studying Caco-2 and LS174-W4 cells, which represent excellent *in vitro* models for polarity studies. Polarity-associated diseases, such as MVID, also serve as useful models to enhance our understanding of the intestinal trafficking and polarity machinery in health and disease. Over the past few years, several mouse models for MVID have been established. It was found that defects in several members of the apical trafficking pathway, such as Rab8a, Rab11a and Cdc42, resulted in some, but not all, hallmarks of human MVID, whereas deletion of *Myo5b* recapitulated MVID completely (Tables 1 and 2). The inducible *Myo5b*-defeicient models can now be used to study the sequence of events on a cellular level in a physiological context, which is not possible with *in vitro* models of genetically manipulated cell lines.

However, the mutations seen in MVID patients are very heterogeneous, and disease phenotypes can vary between individual patients, with some patients even being reported to have episodes where they can tolerate (partial) enteral feeding (Phillips and Schmitz, 1992; Ruemmele et al., 2006). By contrast, all mouse models resulted in a complete loss of function of the MYO5B protein. This underlines the importance to not only rely on *in vitro* or *in vivo* models to elucidate the pathophysiology of MVID, but to combine those models with patient data. Indeed, new genetic mutations causing MVID have been identified recently, and an online MVID patient registry has been established (van der Velde et al., 2013). Furthermore, we foresee that the use of primary *in vitro* models, such as intestinal organoids, will play an important role in intestinal polarity studies, because they contain the patient-specific mutation and can be used to study the epithelial pathophysiological defects. The main advantage of using organoids is that they can be easily established from mouse models as well as from individual patients (Schneeberger et al., 2015; Vogel et al., 2017b; Wiegerinck et al., 2014), and can be infinitely expanded in culture. Whereas the analysis of patient biopsies always represents a static picture of the moment when the biopsy was taken, organoids derived from patients allow for a dynamic analysis of the disease phenotype by functional assays or live cell imaging (Dekkers et al., 2013). Furthermore, the use of organoids enables personalized interventions, such as nutrient challenges, personalized drug screening and drug development (Dekkers et al., 2016).

Currently, MVID diagnosis is dependent on classical histology and (electron) microscopy, while the variability of described features could lead to misinterpretation or incorrect diagnosis of MVID. In addition, there are patients known to present with an MVID phenotype, but do not harbor mutations in *MYO5B*, *STX3* or *STXBP2*, suggesting the involvement of yet unknown genes in causing MVID (S.M., personal communication). Therefore, we suggest the inclusion of standard genetic testing as standard workflow for MVID patients, expansion of the MVID patient registry, and creation and biobanking of patient-specific stem cell-based organoids from MVID patients to allow genotype-to-phenotype comparisons (Box 3). The additional genetic and clinical information in the patient registry will allow a better understanding of the variations in the MVID phenotype, and will provide clinicians with a better overview of the clinical presentations that are related to MVID.

**Box 3. Combined approaches for MVID research, diagnosis and treatment**

Currently, a MVID diagnosis is dependent on classical histology and (electron) microscopy, while the variability of described features could lead to misinterpretation or incorrect diagnosis of MVID. Therefore, we suggest the following combined approach as a standard workflow for MVID patients:

• Standardized genetic testing and extensive automated microscopy to enhance the differential diagnosis of MVID in the clinic.

• The expansion and maintenance of MVID patient registries that integrate basic, genetic and clinical data to allow genotype-to-phenotype comparisons.

• Creating and biobanking patient-derived stem cell-based organoids from MVID patients for disease modeling, drug screening and drug development.

## References

[DMM031088C1] Achler C., Filmer D., Merte C. and Drenckhahn D. (1989). Role of microtubules in polarized delivery of apical membrane proteins to the brush border of the intestinal epithelium. *J. Cell Biol.* 109, 179-189. 10.1083/jcb.109.1.179 2568363 PMC2115479

[DMM031088C2] Akhmanova A. and Hoogenraad C. C. (2015). Microtubule minus-end-targeting proteins. *Curr. Biol.* 25, R162-R171. 10.1016/j.cub.2014.12.027 25689915

[DMM031088C3] Ameen N. and Apodaca G. (2007). Defective CFTR apical endocytosis and enterocyte brush border in myosin VI-deficient mice. *Traffic* 8, 998-1006. 10.1111/j.1600-0854.2007.00587.x 17555536

[DMM031088C4] Ameen N. A. and Salas P. J. I. (2000). Microvillus inclusion disease: a genetic defect affecting apical membrane protein traffic in intestinal epithelium. *Traffic* 1, 76-83. 10.1034/j.1600-0854.2000.010111.x 11208062

[DMM031088C5] Antileo E., Garri C., Tapia V., Muñoz J. P., Chiong M., Nualart F., Lavandero S., Fernández J. and Núñez M. T. (2013). Endocytic pathway of exogenous iron-loaded ferritin in intestinal epithelial (Caco-2) cells. *Am. J. Physiol. Gastrointest. Liver Physiol.* 304, G655-G661. 10.1152/ajpgi.00472.2012 23370673

[DMM031088C6] Apodaca G. (2001). Endocytic traffic in polarized epithelial cells: role of the actin and microtubule cytoskeleton. *Traffic* 2, 149-159. 10.1034/j.1600-0854.2001.020301.x 11260520

[DMM031088C7] Baas A. F., Kuipers J., van der Wel N. N., Batlle E., Koerten H. K., Peters P. J. and Clevers H. C. (2004). Complete polarization of single intestinal epithelial cells upon activation of LKB1 by STRAD. *Cell* 116, 457-466. 10.1016/S0092-8674(04)00114-X 15016379

[DMM031088C8] Bigorgne A. E., Farin H. F., Lemoine R., Mahlaoui N., Lambert N., Gil M., Schulz A., Philippet P., Schlesser P., Abrahamsen T. G. et al. (2014). TTC7A mutations disrupt intestinal epithelial apicobasal polarity. *J. Clin. Invest.* 124, 328-337. 10.1172/JCI71471 24292712 PMC3871247

[DMM031088C9] Blokzijl F., de Ligt J., Jager M., Sasselli V., Roerink S., Sasaki N., Huch M., Boymans S., Kuijk E., Prins P. et al. (2016). Tissue-specific mutation accumulation in human adult stem cells during life. *Nature* 538, 260-264. 10.1038/nature19768 27698416 PMC5536223

[DMM031088C10] Bretscher A., Reczek D. and Berryman M. (1997). Ezrin: a protein requiring conformational activation to link microfilaments to the plasma membrane in the assembly of cell surface structures. *J. Cell Sci.* 110, 3011-3018. 9365271 10.1242/jcs.110.24.3011

[DMM031088C11] Bretscher A., Edwards K. and Fehon R. G. (2002). ERM proteins and merlin: integrators at the cell cortex. *Nat. Rev. Mol. Cell Biol.* 3, 586-599. 10.1038/nrm882 12154370

[DMM031088C12] Breuza L., Fransen J. and Le Bivic A. (2000). Transport and function of syntaxin 3 in human epithelial intestinal cells. *Am. J. Physiol. Cell Physiol.* 279, C1239-C1248. 10.1152/ajpcell.2000.279.4.C1239 11003604

[DMM031088C13] Brown S. S. (1999). Cooperation between microtubule- and actin-based motor proteins. *Annu. Rev. Cell Dev. Biol.* 15, 63-80. 10.1146/annurev.cellbio.15.1.63 10611957

[DMM031088C14] Bryant D. M., Datta A., Rodríguez-Fraticelli A. E., Peränen J., Martín-Belmonte F. and Mostov K. E. (2010). A molecular network for de novo generation of the apical surface and lumen. *Nat. Cell Biol.* 12, 1035-1045. 10.1038/ncb2106 20890297 PMC2975675

[DMM031088C15] Canani R. B., Castaldo G., Bacchetta R., Martín M. G. and Goulet O. (2015). Congenital diarrhoeal disorders: advances in this evolving web of inherited enteropathies. *Nat. Rev. Gastroenterol. Hepatol.* 12, 293-302. 10.1038/nrgastro.2015.44 25782092 PMC7599016

[DMM031088C16] Cartón-García F., Overeem A. W., Nieto R., Bazzocco S., Dopeso H., Macaya I., Bilic J., Landolfi S., Hernandez-Losa J., Schwartz S. Jr. et al. (2015). Myo5b knockout mice as a model of microvillus inclusion disease. *Sci. Rep.* 5, 12312 10.1038/srep12312 26201991 PMC4511872

[DMM031088C17] Casaletto J. B., Saotome I., Curto M. and McClatchey A. I. (2011). Ezrin-mediated apical integrity is required for intestinal homeostasis. *Proc. Natl. Acad. Sci. USA* 108, 11924-11929. 10.1073/pnas.1103418108 21730140 PMC3141968

[DMM031088C18] Chang J., Chance M. R., Nicholas C., Ahmed N., Guilmeau S., Flandez M., Wang D., Byun D.-S., Nasser S., Albanese J. M. et al. (2008). Proteomic changes during intestinal cell maturation in vivo. *J. Proteomics* 71, 530-546. 10.1016/j.jprot.2008.08.003 18824147 PMC2655360

[DMM031088C19] Chantret I., Barbat A., Dussaulx E., Brattain M. G. and Zweibaum A. (1988). Epithelial polarity, villin expression, and enterocytic differentiation of cultured human colon carcinoma cells: a survey of twenty cell lines. *Cancer Res.* 48, 1936-1942. 3349466

[DMM031088C20] Clevers H. (2013). The intestinal crypt, a prototype stem cell compartment. *Cell* 154, 274-284. 10.1016/j.cell.2013.07.004 23870119

[DMM031088C21] Crawley S. W., Mooseker M. S. and Tyska M. J. (2014). Shaping the intestinal brush border. *J. Cell Biol.* 207, 441-451. 10.1083/jcb.201407015 25422372 PMC4242837

[DMM031088C22] Cutz E., Rhoads J. M., Drumm B., Sherman P. M., Durie P. R. and Forstner G. G. (1989). Microvillus inclusion disease: an inherited defect of brush-border assembly and differentiation. *N. Engl. J. Med.* 320, 646-651. 10.1056/NEJM198903093201006 2537465

[DMM031088C23] Cutz E., Sherman P. M. and Davidson G. P. (1997). Enteropathies associated with protracted diarrhea of infancy: clinicopathological features, cellular and molecular mechanisms. *Pediatr. Pathol. Lab. Med.* 17, 335-368. 9185217

[DMM031088C24] Dammermann A., Desai A. and Oegema K. (2003). The minus end in sight. *Curr. Biol.* 13, R614-R624. 10.1016/S0960-9822(03)00530-X 12906817

[DMM031088C25] Danielsen E. M. and Hansen G. H. (2008). Lipid raft organization and function in the small intestinal brush border. *J. Physiol. Biochem.* 64, 377-382. 10.1007/BF03174093 19391463

[DMM031088C26] Davidson G. P., Cutz E., Hamilton J. R. and Gall D. G. (1978). Familial enteropathy: a syndrome of protracted diarrhea from birth, failure to thrive, and hypoplastic villus atrophy. *Gastroenterology* 75, 783-790. 100367

[DMM031088C27] Dekkers J. F., Wiegerinck C. L., de Jonge H. R., Bronsveld I., Janssens H. M., de Winter-de Groot K. M., Brandsma A. M., de Jong N. W. M., Bijvelds M. J. C., Scholte B. J. et al. (2013). A functional CFTR assay using primary cystic fibrosis intestinal organoids. *Nat. Med.* 19, 939-945. 10.1038/nm.3201 23727931

[DMM031088C28] Dekkers J. F., Berkers G., Kruisselbrink E., Vonk A., de Jonge H. R., Janssens H. M., Bronsveld I., van de Graaf E. A., Nieuwenhuis E. E., Houwen R. H. et al. (2016). Characterizing responses to CFTR-modulating drugs using rectal organoids derived from subjects with cystic fibrosis. *Sci. Transl. Med.* 8, 344ra84 10.1126/scitranslmed.aad8278 27334259

[DMM031088C29] Delgrossi M. H., Breuza L., Mirre C., Chavrier P. and Le Bivic A. (1997). Human syntaxin 3 is localized apically in human intestinal cells. *J. Cell Sci.* 110, 2207-2214. 9378770 10.1242/jcs.110.18.2207

[DMM031088C30] Dhekne H. S., Hsiao N.-H., Roelofs P., Kumari M., Slim C. L., Rings E. H. H. M. and van Ijzendoorn S. C. D. (2014). Myosin Vb and Rab11a regulate phosphorylation of ezrin in enterocytes. *J. Cell Sci.* 127, 1007-1017. 10.1242/jcs.137273 24413175

[DMM031088C31] Farquhar M. G. and Palade G. E. (1963). Junctional complexes in various epithelia. *J. Cell Biol.* 17, 375-412. 10.1083/jcb.17.2.375 13944428 PMC2106201

[DMM031088C32] Fatehullah A., Appleton P. L. and Nathke I. S. (2013). Cell and tissue polarity in the intestinal tract during tumourigenesis: cells still know the right way up, but tissue organization is lost. *Philos. Trans. R. Soc. Lond. B Biol. Sci.* 368, 20130014 10.1098/rstb.2013.0014 24062584 PMC3785964

[DMM031088C33] Fehon R. G., McClatchey A. I. and Bretscher A. (2010). Organizing the cell cortex: the role of ERM proteins. *Nat. Rev. Mol. Cell Biol.* 11, 276-287. 10.1038/nrm2866 20308985 PMC2871950

[DMM031088C34] Feng Q., Bonder E. M., Engevik A. C., Zhang L., Tyska M. J., Goldenring J. R. and Gao N. (2017). Disruption of Rab8a and Rab11a causes formation of basolateral microvilli in neonatal enteropathy. *J. Cell Sci.* 130, 2491-2505. 10.1242/jcs.201897 28596241 PMC5558269

[DMM031088C35] Ferrary E., Cohen-Tannoudji M., Pehau-Arnaudet G., Lapillonne A., Athman R., Ruiz T., Boulouha L., El Marjou F., Doye A., Fontaine J.-J. et al. (1999). In vivo, villin is required for Ca(2+)-dependent F-actin disruption in intestinal brush borders. *J. Cell Biol.* 146, 819-830. 10.1083/jcb.146.4.819 10459016 PMC2156144

[DMM031088C36] Fleet J. C., Wang L., Vitek O., Craig B. A. and Edenberg H. J. (2003). Gene expression profiling of Caco-2 BBe cells suggests a role for specific signaling pathways during intestinal differentiation. *Physiol. Genomics* 13, 57-68. 10.1152/physiolgenomics.00152.2002 12644633

[DMM031088C37] Fujita M., Reinhart F. and Neutra M. (1990). Convergence of apical and basolateral endocytic pathways at apical late endosomes in absorptive cells of suckling rat ileum in vivo. *J. Cell Sci.* 97, 385-394. 2277098 10.1242/jcs.97.2.385

[DMM031088C38] Galli T., Zahraoui A., Vaidyanathan V. V., Raposo G., Tian J. M., Karin M., Niemann H. and Louvard D. (1998). A novel tetanus neurotoxin-insensitive vesicle-associated membrane protein in SNARE complexes of the apical plasma membrane of epithelial cells. *Mol. Biol. Cell* 9, 1437-1448. 10.1091/mbc.9.6.1437 9614185 PMC25366

[DMM031088C39] Giepmans B. N. G. and van Ijzendoorn S. C. D. (2009). Epithelial cell-cell junctions and plasma membrane domains. *Biochim. Biophys. Acta* 1788, 820-831. 10.1016/j.bbamem.2008.07.015 18706883

[DMM031088C40] Gilbert T., Le Bivic A., Quaroni A. and Rodriguez-Boulan E. (1991). Microtubular organization and its involvement in the biogenetic pathways of plasma membrane proteins in Caco-2 intestinal epithelial cells. *J. Cell Biol.* 113, 275-288. 10.1083/jcb.113.2.275 1672691 PMC2288937

[DMM031088C41] Gloerich M., ten Klooster J. P., Vliem M. J., Koorman T., Zwartkruis F. J., Clevers H. and Bos J. L. (2012). Rap2A links intestinal cell polarity to brush border formation. *Nat. Cell Biol.* 14, 793-801. 10.1038/ncb2537 22797597

[DMM031088C42] Golachowska M. R., Hoekstra D. and van IJzendoorn S. C. D. (2010). Recycling endosomes in apical plasma membrane domain formation and epithelial cell polarity. *Trends Cell Biol.* 20, 618-626. 10.1016/j.tcb.2010.08.004 20833047

[DMM031088C43] Grimm-Gunter E.-M. S., Revenu C., Ramos S., Hurbain I., Smyth N., Ferrary E., Louvard D., Robine S. and Rivero F. (2009). Plastin 1 binds to keratin and is required for terminal web assembly in the intestinal epithelium. *Mol. Biol. Cell* 20, 2549-2562. 10.1091/mbc.E08-10-1030 19321664 PMC2682596

[DMM031088C44] Hales C. M., Vaerman J.-P. and Goldenring J. R. (2002). Rab11 family interacting protein 2 associates with Myosin Vb and regulates plasma membrane recycling. *J. Biol. Chem.* 277, 50415-50421. 10.1074/jbc.M209270200 12393859

[DMM031088C45] Hegan P. S., Giral H., Levi M. and Mooseker M. S. (2012). Myosin VI is required for maintenance of brush border structure, composition, and membrane trafficking functions in the intestinal epithelial cell. *Cytoskeleton (Hoboken)* 69, 235-251. 10.1002/cm.21018 22328452 PMC3328626

[DMM031088C46] Heintzelman M. B. and Mooseker M. S. (1992). Assembly of the intestinal brush border cytoskeleton. *Curr. Top. Dev. Biol.* 26, 93-122. 10.1016/S0070-2153(08)60442-1 1563281

[DMM031088C47] Heintzelman M. B., Hasson T. and Mooseker M. S. (1994). Multiple unconventional myosin domains of the intestinal brush border cytoskeleton. *J. Cell Sci.* 107, 3535-3543. 7706404 10.1242/jcs.107.12.3535

[DMM031088C48] Hong W. J. and Lev S. (2014). Tethering the assembly of SNARE complexes. *Trends Cell Biol.* 24, 35-43. 10.1016/j.tcb.2013.09.006 24119662

[DMM031088C49] Iancu T. C., Mahajnah M., Manov I. and Shaoul R. (2007). Microvillous inclusion disease: ultrastructural variability. *Ultrastruct. Pathol.* 31, 173-188. 10.1080/01913120701350712 17613997

[DMM031088C50] Jacob R. and Naim H. Y. (2001). Apical membrane proteins are transported in distinct vesicular carriers. *Curr. Biol.* 11, 1444-1450. 10.1016/S0960-9822(01)00446-8 11566104

[DMM031088C51] Kahan B. D., Rutzky L., Berlin B., Tomita J., Wiseman F., LeGrue S., Noll H. and Tom B. H. (1976). Cell surface alterations on colon adenocarcinoma cells. *Cancer Res.* 36, 3526-3534. 975112

[DMM031088C52] Kapitein L. C., van Bergeijk P., Lipka J., Keijzer N., Wulf P. S., Katrukha E. A., Akhmanova A. and Hoogenraad C. C. (2013). Myosin-V opposes microtubule-based cargo transport and drives directional motility on cortical actin. *Curr. Biol.* 23, 828-834. 10.1016/j.cub.2013.03.068 23602478

[DMM031088C141] Keiser M., Alfalah M., Propsting M. J., Castelletti D. and Naim H. Y. (2006). Altered folding, turnover, and polarized sorting act in concert to define a novel pathomechanism of congenital sucrase-isomaltase deficiency. *J. Biol. Chem.* 281, 14393-14399. 10.1074/jbc.M513631200 16543230

[DMM031088C53] Klunder L. J., Faber K. N., Dijkstra G. and van Ijzendoorn S. C. D. (2017). Mechanisms of Cell Polarity-Controlled Epithelial Homeostasis and Immunity in the Intestine. *Cold Spring Harb. Perspect. Biol.* 9, a027888 10.1101/cshperspect.a027888 28213466 PMC5495056

[DMM031088C54] Knowles B. C., Roland J. T., Krishnan M., Tyska M. J., Lapierre L. A., Dickman P. S., Goldenring J. R. and Shub M. D. (2014). Myosin Vb uncoupling from RAB8A and RAB11A elicits microvillus inclusion disease. *J. Clin. Invest.* 124, 2947-2962. 10.1172/JCI71651 24892806 PMC4071383

[DMM031088C55] Knowles B. C., Weis V. G., Yu S., Roland J. T., Williams J. A., Alvarado G. S., Lapierre L. A., Shub M. D., Gao N. and Goldenring J. R. (2015). Rab11a regulates syntaxin 3 localization and microvillus assembly in enterocytes. *J. Cell Sci.* 128, 1617-1626. 10.1242/jcs.163303 25673875 PMC4518445

[DMM031088C56] Koepsell S. A. and Talmon G. (2010). Light microscopic diagnosis of microvillus inclusion disease on colorectal specimens using CD10. *Am. J. Surg. Pathol.* 34, 970-972. 10.1097/PAS.0b013e3181e11e4b 20505500

[DMM031088C57] Koo B.-K., Stange D. E., Sato T., Karthaus W., Farin H. F., Huch M., van Es J. H. and Clevers H. (2012). Controlled gene expression in primary Lgr5 organoid cultures. *Nat. Methods* 9, 81-83. 10.1038/nmeth.1802 22138822

[DMM031088C58] Koo B.-K., Sasselli V. and Clevers H. (2013). Retroviral gene expression control in primary organoid cultures. *Curr. Protoc. Stem Cell Biol.* 27, 5A.6.1-5A.6.8 10.1002/9780470151808.sc05a06s27 24510288

[DMM031088C59] Kravtsov D. V. and Ameen N. A. (2013). Molecular motors and apical CFTR traffic in epithelia. *Int. J. Mol. Sci.* 14, 9628-9642. 10.3390/ijms14059628 23644890 PMC3676803

[DMM031088C60] Kravtsov D., Mashukova A., Forteza R., Rodriguez M. M., Ameen N. A. and Salas P. J. (2014). Myosin 5b loss of function leads to defects in polarized signaling: implication for microvillus inclusion disease pathogenesis and treatment. *Am. J. Physiol. Gastrointest. Liver Physiol.* 307, G992-G1001. 10.1152/ajpgi.00180.2014 25258405 PMC4233287

[DMM031088C61] Kravtsov D. V., Ahsan M. K., Kumari V., van Ijzendoorn S. C. D., Reyes-Mugica M., Kumar A., Gujral T., Dudeja P. K. and Ameen N. A. (2016). Identification of intestinal ion transport defects in microvillus inclusion disease. *Am. J. Physiol. Gastrointest. Liver Physiol.* 311, G142-G155. 10.1152/ajpgi.00041.2016 27229121 PMC4967175

[DMM031088C62] Lake B. D. (1988). Microvillus inclusion disease: specific diagnostic features shown by alkaline phosphatase histochemistry. *J. Clin. Pathol.* 41, 880-882. 10.1136/jcp.41.8.880 3170775 PMC1141620

[DMM031088C63] Lapierre L. A., Kumar R., Hales C. M., Navarre J., Bhartur S. G., Burnette J. O., Provance D. W. Jr. Mercer J. A., Bahler M. and Goldenring J. R. (2001). Myosin vb is associated with plasma membrane recycling systems. *Mol. Biol. Cell* 12, 1843-1857. 10.1091/mbc.12.6.1843 11408590 PMC37346

[DMM031088C64] Laukoetter M. G., Bruewer M. and Nusrat A. (2006). Regulation of the intestinal epithelial barrier by the apical junctional complex. *Curr. Opin Gastroenterol.* 22, 85-89. 10.1097/01.mog.0000203864.48255.4f 16462161

[DMM031088C65] Le Bivic A., Quaroni A., Nichols B. and Rodriguez-Boulan E. (1990). Biogenetic pathways of plasma membrane proteins in Caco-2, a human intestinal epithelial cell line. *J. Cell Biol.* 111, 1351-1361. 10.1083/jcb.111.4.1351 1976637 PMC2116246

[DMM031088C66] Maroux S., Coudrier E., Feracci H., Gorvel J.-P. and Louvard D. (1988). Molecular organization of the intestinal brush border. *Biochimie* 70, 1297-1306. 10.1016/0300-9084(88)90198-8 3147722

[DMM031088C67] Massey-Harroche D. (2000). Epithelial cell polarity as reflected in enterocytes. *Microsc. Res. Tech.* 49, 353-362. 10.1002/(SICI)1097-0029(20000515)49:4<353::AID-JEMT4>3.0.CO;2-8 10820519

[DMM031088C68] Matter K., Brauchbar M., Bucher K. and Hauri H.-P. (1990). Sorting of endogenous plasma membrane proteins occurs from two sites in cultured human intestinal epithelial cells (Caco-2). *Cell* 60, 429-437. 10.1016/0092-8674(90)90594-5 2302734

[DMM031088C69] Mazerik J. N. and Tyska M. J. (2012). Myosin-1A targets to microvilli using multiple membrane binding motifs in the tail homology 1 (TH1) domain. *J. Biol. Chem.* 287, 13104-13115. 10.1074/jbc.M111.336313 22367206 PMC3339983

[DMM031088C70] McNiven M. A. and Marlowe K. J. (1999). Contributions of molecular motor enzymes to vesicle-based protein transport in gastrointestinal epithelial cells. *Gastroenterology* 116, 438-451. 10.1016/S0016-5085(99)70142-3 9922326

[DMM031088C71] Melendez J., Liu M., Sampson L., Akunuru S., Han X., Vallance J., Witte D., Shroyer N. and Zheng Y. (2013). Cdc42 coordinates proliferation, polarity, migration, and differentiation of small intestinal epithelial cells in mice. *Gastroenterology* 145, 808-819. 10.1053/j.gastro.2013.06.021 23792201 PMC3876942

[DMM031088C72] Mendes C., Figueiredo C., Mansilha H., Proenca E., Oliveira D., Lima R. and Carvalho C. (2014). A case of protracted diarrhea in a newborn: a diagnostic challenge. *Pediatr. Rep.* 6, 5596 10.4081/pr.2014.5596 25635218 PMC4292062

[DMM031088C73] Michail S., Collins J. F., Xu H., Kaufman S., Vanderhoof J. and Ghishan F. K. (1998). Abnormal expression of brush-border membrane transporters in the duodenal mucosa of two patients with microvillus inclusion disease. *J. Pediatr. Gastroenterol. Nutr.* 27, 536-542. 10.1097/00005176-199811000-00008 9822319

[DMM031088C140] Michaux G., Massey-Harroche D., Nicolle O., Rabant M., Brousse N., Goulet O., Le Bivic A. and Ruemmele F. M. (2016). The localisation of the apical Par/Cdc42 polarity module is specifically affected in microvillus inclusion disease. *Biol. Cell* 108, 19-28. 10.1111/boc.201500034 26526116

[DMM031088C74] Middendorp S., Schneeberger K., Wiegerinck C. L., Mokry M., Akkerman R. D. L., van Wijngaarden S., Clevers H. and Nieuwenhuis E. E. S. (2014). Adult stem cells in the small intestine are intrinsically programmed with their location-specific function. *Stem Cells* 32, 1083-1091. 10.1002/stem.1655 24496776

[DMM031088C75] Mierau G. W., Wills E. J., Wyatt-Ashmead J., Hoffenberg E. J. and Cutz E. (2001). Microvillous inclusion disease: report of a case with atypical features. *Ultrastruct. Pathol.* 25, 275-279. 10.1080/01913120119148 11465482

[DMM031088C76] Moon C., VanDussen K. L., Miyoshi H. and Stappenbeck T. S. (2014). Development of a primary mouse intestinal epithelial cell monolayer culture system to evaluate factors that modulate IgA transcytosis. *Mucosal. Immunol.* 7, 818-828. 10.1038/mi.2013.98 24220295 PMC4019725

[DMM031088C77] Mooseker M. S. (1985). Organization, chemistry, and assembly of the cytoskeletal apparatus of the intestinal brush border. *Annu. Rev. Cell Biol.* 1, 209-241. 10.1146/annurev.cb.01.110185.001233 3916317

[DMM031088C78] Mukherjee S., Vaishnava S. and Hooper L. V. (2008). Multi-layered regulation of intestinal antimicrobial defense. *Cell. Mol. Life Sci.* 65, 3019-3027. 10.1007/s00018-008-8182-3 18560756 PMC11131881

[DMM031088C79] Müller T., Hess M. W., Schiefermeier N., Pfaller K., Ebner H. L., Heinz-Erian P., Ponstingl H., Partsch J., Röllinghoff B., Köhler H. et al. (2008). MYO5B mutations cause microvillus inclusion disease and disrupt epithelial cell polarity. *Nat. Genet.* 40, 1163-1165. 10.1038/ng.225 18724368

[DMM031088C80] Noordstra I., Liu Q., Nijenhuis W., Hua S., Jiang K., Baars M., Remmelzwaal S., Martin M., Kapitein L. C. and Akhmanova A. (2016). Control of apico-basal epithelial polarity by the microtubule minus-end-binding protein CAMSAP3 and spectraplakin ACF7. *J. Cell Sci.* 129, 4278-4288. 10.1242/jcs.194878 27802168

[DMM031088C81] Overeem A. W., Posovszky C., Rings E. H. M. M., Giepmans B. N. G. and van IJzendoorn S. C. D. (2016). The role of enterocyte defects in the pathogenesis of congenital diarrheal disorders. *Dis. Model. Mech.* 9, 1-12. 10.1242/dmm.022269 26747865 PMC4728335

[DMM031088C82] Peterson M. D. and Mooseker M. S. (1992). Characterization of the enterocyte-like brush border cytoskeleton of the C2BBe clones of the human intestinal cell line, Caco-2. *J. Cell Sci.* 102, 581-600. 1506435 10.1242/jcs.102.3.581

[DMM031088C83] Peterson M. D. and Mooseker M. S. (1993). An in vitro model for the analysis of intestinal brush border assembly. I. Ultrastructural analysis of cell contact-induced brush border assembly in Caco-2BBe cells. *J. Cell Sci.* 105, 445-460. 8408276 10.1242/jcs.105.2.445

[DMM031088C84] Peterson M. D., Bement W. M. and Mooseker M. S. (1993). An in vitro model for the analysis of intestinal brush border assembly. II. Changes in expression and localization of brush border proteins during cell contact-induced brush border assembly in Caco-2BBe cells. *J. Cell Sci.* 105, 461-472. 8408277 10.1242/jcs.105.2.461

[DMM031088C85] Phillips A. D. and Schmitz J. (1992). Familial microvillous atrophy: a clinicopathological survey of 23 cases. *J. Pediatr. Gastroenterol. Nutr.* 14, 380-396. 10.1097/00005176-199205000-00003 1355534

[DMM031088C86] Phillips A. D., Szafranski M., Man L.-Y. and Wall W. J. (2000). Periodic acid-Schiff staining abnormality in microvillous atrophy: photometric and ultrastructural studies. *J. Pediatr. Gastroenterol. Nutr.* 30, 34-42. 10.1097/00005176-200001000-00015 10630437

[DMM031088C87] Pocard T., Le Bivic A., Galli T. and Zurzolo C. (2007). Distinct v-SNAREs regulate direct and indirect apical delivery in polarized epithelial cells. *J. Cell Sci.* 120, 3309-3320. 10.1242/jcs.007948 17878240

[DMM031088C88] Pohl J. F., Shub M. D., Trevelline E. E., Ingebo K., Silber G., Rayhorn N., Holve S. and Hu D. (1999). A cluster of microvillous inclusion disease in the Navajo population. *J. Pediatr.* 134, 103-106. 10.1016/S0022-3476(99)70380-X 9880458

[DMM031088C89] Raafat F., Green N. J., Nathavitharana K. A. and Booth I. W. (1994). Intestinal microvillous dystrophy: a variant of microvillous inclusion disease or a new entity? *Hum. Pathol.* 25, 1243-1248. 10.1016/0046-8177(94)90043-4 7959671

[DMM031088C90] Rapetti-Mauss R., O'Mahony F., Sepulveda F. V., Urbach V. and Harvey B. J. (2013). Oestrogen promotes KCNQ1 potassium channel endocytosis and postendocytic trafficking in colonic epithelium. *J. Physiol.* 591, 2813-2831. 10.1113/jphysiol.2013.251678 23529131 PMC3690688

[DMM031088C91] Reinshagen K., Naim H. Y. and Zimmer K. P. (2002). Autophagocytosis of the apical membrane in microvillus inclusion disease. *Gut* 51, 514-521. 10.1136/gut.51.4.514 12235073 PMC1773396

[DMM031088C92] Revenu C., Ubelmann F., Hurbain I., El-Marjou F., Dingli F., Loew D., Delacour D., Gilet J., Brot-Laroche E., Rivero F. et al. (2012). A new role for the architecture of microvillar actin bundles in apical retention of membrane proteins. *Mol. Biol. Cell* 23, 324-336. 10.1091/mbc.E11-09-0765 22114352 PMC3258176

[DMM031088C93] Rhoads J. M., Vogler R. C., Lacey S. R., Reddick R. L., Keku E. O., Azizkhan R. G. and Berschneider H. M. (1991). Microvillus inclusion disease. In vitro jejunal electrolyte transport. *Gastroenterology* 100, 811-817. 10.1016/0016-5085(91)80031-4 1993505

[DMM031088C94] Riento K., Galli T., Jansson S., Ehnholm C., Lehtonen E. and Olkkonen V. M. (1998). Interaction of Munc-18-2 with syntaxin 3 controls the association of apical SNAREs in epithelial cells. *J. Cell Sci.* 111, 2681-2688. 9701566 10.1242/jcs.111.17.2681

[DMM031088C95] Rodriguez-Boulan E., Kreitzer G. and Müsch A. (2005). Organization of vesicular trafficking in epithelia. *Nat. Rev. Mol. Cell Biol.* 6, 233-247. 10.1038/nrm1593 15738988

[DMM031088C96] Roland J. T., Kenworthy A. K., Peranen J., Caplan S. and Goldenring J. R. (2007). Myosin Vb interacts with Rab8a on a tubular network containing EHD1 and EHD3. *Mol. Biol. Cell* 18, 2828-2837. 10.1091/mbc.E07-02-0169 17507647 PMC1949367

[DMM031088C97] Roland J. T., Bryant D. M., Datta A., Itzen A., Mostov K. E. and Goldenring J. R. (2011). Rab GTPase-Myo5B complexes control membrane recycling and epithelial polarization. *Proc. Natl. Acad. Sci. USA* 108, 2789-2794. 10.1073/pnas.1010754108 21282656 PMC3041130

[DMM031088C98] Ruemmele F. M., Schmitz J. and Goulet O. (2006). Microvillous inclusion disease (microvillous atrophy). *Orphanet J. Rare Dis.* 1, 22 10.1186/1750-1172-1-22 16800870 PMC1523325

[DMM031088C99] Ruemmele F. M., Müller T., Schiefermeier N., Ebner H. L., Lechner S., Pfaller K., Thöni C. E., Goulet O., Lacaille F., Schmitz J. et al. (2010). Loss-of-function of MYO5B is the main cause of microvillus inclusion disease: 15 novel mutations and a CaCo-2 RNAi cell model. *Hum. Mutat.* 31, 544-551. 10.1002/humu.21224 20186687

[DMM031088C100] Sakamori R., Das S., Yu S., Feng S., Stypulkowski E., Guan Y., Douard V., Tang W., Ferraris R. P., Harada A. et al. (2012). Cdc42 and Rab8a are critical for intestinal stem cell division, survival, and differentiation in mice. *J. Clin. Invest.* 122, 1052-1065. 10.1172/JCI60282 22354172 PMC3287229

[DMM031088C101] Sandoz D., Laine M. C. and Nicolas G. (1986). Distribution of microtubules within the intestinal terminal web as revealed by quick-freezing and cryosubstitution. *Eur. J. Cell Biol.* 39, 481-484. 3956519

[DMM031088C102] Saotome I., Curto M. and McClatchey A. I. (2004). Ezrin is essential for epithelial organization and villus morphogenesis in the developing intestine. *Dev. Cell* 6, 855-864. 10.1016/j.devcel.2004.05.007 15177033

[DMM031088C103] Sato T., Mushiake S., Kato Y., Sato K., Sato M., Takeda N., Ozono K., Miki K., Kubo Y., Tsuji A. et al. (2007). The Rab8 GTPase regulates apical protein localization in intestinal cells. *Nature* 448, 366-369. 10.1038/nature05929 17597763

[DMM031088C104] Sato T., Vries R. G., Snippert H. J., van de Wetering M., Barker N., Stange D. E., van Es J. H., Abo A., Kujala P., Peters P. J. et al. (2009). Single Lgr5 stem cells build crypt-villus structures in vitro without a mesenchymal niche. *Nature* 459, 262-265. 10.1038/nature07935 19329995

[DMM031088C105] Sato T., Stange D. E., Ferrante M., Vries R. G. J., Van Es J. H., Van den Brink S., Van Houdt W. J., Pronk A., Van Gorp J., Siersema P. D. et al. (2011). Long-term expansion of epithelial organoids from human colon, adenoma, adenocarcinoma, and Barrett's epithelium. *Gastroenterology* 141, 1762-1772. 10.1053/j.gastro.2011.07.050 21889923

[DMM031088C106] Schneeberger K., Vogel G. F., Teunissen H., van Ommen D. D., Begthel H., El Bouazzaoui L., van Vugt A. H. M., Beekman J. M., Klumperman J., Müller T. et al. (2015). An inducible mouse model for microvillus inclusion disease reveals a role for myosin Vb in apical and basolateral trafficking. *Proc. Natl. Acad. Sci. USA* 112, 12408-12413. 10.1073/pnas.1516672112 26392529 PMC4603458

[DMM031088C107] Schwank G., Andersson-Rolf A., Koo B.-K., Sasaki N. and Clevers H. (2013a). Generation of BAC transgenic epithelial organoids. *PLoS ONE* 8, e76871 10.1371/journal.pone.0076871 24204693 PMC3800075

[DMM031088C108] Schwank G., Koo B.-K., Sasselli V., Dekkers J. F., Heo I., Demircan T., Sasaki N., Boymans S., Cuppen E., van der Ent C. K. et al. (2013b). Functional repair of CFTR by CRISPR/Cas9 in intestinal stem cell organoids of cystic fibrosis patients. *Cell Stem Cell* 13, 653-658. 10.1016/j.stem.2013.11.002 24315439

[DMM031088C109] Shahid S., Fraser D. D., Driman D. K. and Bax K. C. (2012). Severe hypernatremic dehydration and metabolic acidosis due to neonatal intestinal microvillus inclusion disease. *Neonatology* 101, 154-158. 10.1159/000330570 21968248

[DMM031088C110] Shen L., Weber C. R., Raleigh D. R., Yu D. and Turner J. R. (2011). Tight junction pore and leak pathways: a dynamic duo. *Annu. Rev. Physiol.* 73, 283-309. 10.1146/annurev-physiol-012110-142150 20936941 PMC4655434

[DMM031088C111] Sherman P. M., Mitchell D. J. and Cutz E. (2004). Neonatal enteropathies: defining the causes of protracted diarrhea of infancy. *J. Pediatr. Gastroenterol. Nutr.* 38, 16-26. 10.1097/00005176-200401000-00007 14676590

[DMM031088C112] Shifrin D. A. Jr. and Tyska M. J. (2012). Ready…aim…fire into the lumen: a new role for enterocyte microvilli in gut host defense. *Gut Microbes* 3, 460-462. 10.4161/gmic.21247 22825496 PMC3466500

[DMM031088C113] Shillingford N. M., Calicchio M. L., Teot L. A., Boyd T., Kurek K. C., Goldsmith J. D., Bousvaros A., Perez-Atayde A. R. and Kozakewich H. P. W. (2015). Villin immunohistochemistry is a reliable method for diagnosing microvillus inclusion disease. *Am. J. Surg. Pathol.* 39, 245-250. 10.1097/PAS.0000000000000355 25517957

[DMM031088C114] Sidhaye J., Pinto C. S., Dharap S., Jacob T., Bhargava S. and Sonawane M. (2016). The zebrafish goosepimples/myosin Vb mutant exhibits cellular attributes of human microvillus inclusion disease. *Mech. Dev.* 142, 62-74. 10.1016/j.mod.2016.08.001 27497746 PMC5161235

[DMM031088C115] Smith W. J., Nassar N., Bretscher A., Cerione R. A. and Karplus P. A. (2003). Structure of the active N-terminal domain of Ezrin. Conformational and mobility changes identify keystone interactions. *J. Biol. Chem.* 278, 4949-4956. 10.1074/jbc.M210601200 12429733

[DMM031088C116] Sobajima T., Yoshimura S.-I., Iwano T., Kunii M., Watanabe M., Atik N., Mushiake S., Morii E., Koyama Y., Miyoshi E. et al. (2014). Rab11a is required for apical protein localisation in the intestine. *Biol. Open* 4, 86-94. 10.1242/bio.20148532 25527643 PMC4295169

[DMM031088C117] Somsel Rodman J. and Wandinger-Ness A. (2000). Rab GTPases coordinate endocytosis. *J. Cell Sci.* 113, 183-192. 10633070 10.1242/jcs.113.2.183

[DMM031088C118] Stepensky P., Bartram J., Barth T. F., Lehmberg K., Walther P., Amann K., Philips A. D., Beringer O., Zur Stadt U., Schulz A. et al. (2013). Persistent defective membrane trafficking in epithelial cells of patients with familial hemophagocytic lymphohistiocytosis type 5 due to STXBP2/MUNC18-2 mutations. *Pediatr. Blood Cancer* 60, 1215-1222. 10.1002/pbc.24475 23382066

[DMM031088C119] Straussberg R., Shapiro R., Amir J., Yonash A., Rachmel A., Bisset W. M. and Varsano I. (1997). Congenital intractable diarrhea of infancy in Iraqi Jews. *Clin. Genet.* 51, 98-101. 10.1111/j.1399-0004.1997.tb02428.x 9111996

[DMM031088C120] Stutzmann J., Bellissent-Waydelich A., Fontao L., Launay J.-F. and Simon-Assmann P. (2000). Adhesion complexes implicated in intestinal epithelial cell-matrix interactions. *Microsc. Res. Tech.* 51, 179-190. 10.1002/1097-0029(20001015)51:2<179::AID-JEMT9>3.0.CO;2-4 11054868

[DMM031088C121] Szperl A. M., Golachowska M. R., Bruinenberg M., Prekeris R., Thunnissen A.-M. W. H., Karrenbeld A., Dijkstra G., Hoekstra D., Mercer D., Ksiazyk J. et al. (2011). Functional characterization of mutations in the myosin Vb gene associated with microvillus inclusion disease. *J. Pediatr. Gastroenterol. Nutr.* 52, 307-313. 10.1097/MPG.0b013e3181eea177 21206382 PMC3058815

[DMM031088C122] Talmon G., Holzapfel M., DiMaio D. J. and Muirhead D. (2012). Rab11 is a useful tool for the diagnosis of microvillous inclusion disease. *Int. J. Surg. Pathol.* 20, 252-256. 10.1177/1066896911430959 22169970

[DMM031088C123] ten Klooster J. P., Jansen M., Yuan J., Oorschot V., Begthel H., Di Giacomo V., Colland F., de Koning J., Maurice M. M., Hornbeck P. et al. (2009). Mst4 and Ezrin induce brush borders downstream of the Lkb1/Strad/Mo25 polarization complex. *Dev. Cell* 16, 551-562. 10.1016/j.devcel.2009.01.016 19386264

[DMM031088C124] Thoeni C. E., Vogel G. F., Tancevski I., Geley S., Lechner S., Pfaller K., Hess M. W., Muller T., Janecke A. R., Avitzur Y. et al. (2013). Microvillus inclusion disease: loss of myosin Vb disrupts intracellular traffic and cell polarity. *Traffic* 15, 22-42. 10.1111/tra.12131 24138727

[DMM031088C125] Tocchetti A., Ekalle Soppo C. B., Zani F., Bianchi F., Gagliani M. C., Pozzi B., Rozman J., Elvert R., Ehrhardt N., Rathkolb B. et al. (2010). Loss of the actin remodeler Eps8 causes intestinal defects and improved metabolic status in mice. *PLoS ONE* 5, e9468 10.1371/journal.pone.0009468 20209148 PMC2830459

[DMM031088C126] Toya M., Kobayashi S., Kawasaki M., Shioi G., Kaneko M., Ishiuchi T., Misaki K., Meng W. and Takeichi M. (2016). CAMSAP3 orients the apical-to-basal polarity of microtubule arrays in epithelial cells. *Proc. Natl. Acad. Sci. USA* 113, 332-337. 10.1073/pnas.1520638113 26715742 PMC4720291

[DMM031088C127] Tyska M. J., Mackey A. T., Huang J. D., Copeland N. G., Jenkins N. A. and Mooseker M. S. (2005). Myosin-1a is critical for normal brush border structure and composition. *Mol. Biol. Cell* 16, 2443-2457. 10.1091/mbc.E04-12-1116 15758024 PMC1087248

[DMM031088C128] Utech M., Mennigen R. and Bruewer M. (2010). Endocytosis and recycling of tight junction proteins in inflammation. *J. Biomed. Biotechnol.* 2010, 484987 10.1155/2010/484987 20011071 PMC2789582

[DMM031088C129] van der Velde K. J., Dhekne H. S., Swertz M. A., Sirigu S., Ropars V., Vinke P. C., Rengaw T., van den Akker P. C., Rings E. H. H. M., Houdusse A. et al. (2013). An overview and online registry of microvillus inclusion disease patients and their MYO5B mutations. *Hum. Mutat.* 34, 1597-1605. 10.1002/humu.22440 24014347

[DMM031088C130] VanDussen K. L., Marinshaw J. M., Shaikh N., Miyoshi H., Moon C., Tarr P. I., Ciorba M. A. and Stappenbeck T. S. (2014). Development of an enhanced human gastrointestinal epithelial culture system to facilitate patient-based assays. *Gut* 64, 911-920. 10.1136/gutjnl-2013-306651 25007816 PMC4305344

[DMM031088C131] Viswanatha R., Ohouo P. Y., Smolka M. B. and Bretscher A. (2012). Local phosphocycling mediated by LOK/SLK restricts ezrin function to the apical aspect of epithelial cells. *J. Cell Biol.* 199, 969-984. 10.1083/jcb.201207047 23209304 PMC3518218

[DMM031088C132] Vogel G. F., Klee K. M. C., Janecke A. R., Müller T., Hess M. W. and Huber L. A. (2015). Cargo-selective apical exocytosis in epithelial cells is conducted by Myo5B, Slp4a, Vamp7, and Syntaxin 3. *J. Cell Biol.* 211, 587-604. 10.1083/jcb.201506112 26553929 PMC4639860

[DMM031088C133] Vogel G. F., Janecke A. R., Krainer I. M., Gutleben K., Witting B., Mitton S. G., Mansour S., Ballauff A., Roland J. T., Engevik A. C. et al. (2017a). Abnormal Rab11-Rab8-vesicles cluster in enterocytes of patients with microvillus inclusion disease. *Traffic* 18, 453-464. 10.1111/tra.12486 28407399 PMC5693299

[DMM031088C134] Vogel G. F., van Rijn J. M., Krainer I. M., Janecke A. R., Posovzsky C., Cohen M., Searle C., Jantchou P., Escher J. C., Patey N. et al. (2017b). Disrupted apical exocytosis of cargo vesicles causes enteropathy in FHL5 patients with Munc18-2 mutations. *JCI Insight* 2, e94564 10.1172/jci.insight.94564 PMC5518552 28724787

[DMM031088C135] Wald F. A., Oriolo A. S., Mashukova A., Fregien N. L., Langshaw A. H. and Salas P. J. I. (2008). Atypical protein kinase C (iota) activates ezrin in the apical domain of intestinal epithelial cells. *J. Cell Sci.* 121, 644-654. 10.1242/jcs.016246 18270268 PMC2293289

[DMM031088C136] Weis V. G., Knowles B. C., Choi E., Goldstein A. E., Williams J. A., Manning E. H., Roland J. T., Lapierre L. A. and Goldenring J. R. (2016). Loss of MYO5B in mice recapitulates Microvillus Inclusion Disease and reveals an apical trafficking pathway distinct to neonatal duodenum. *Cell Mol. Gastroenterol. Hepatol.* 2, 131-157. 10.1016/j.jcmgh.2015.11.009 27019864 PMC4806369

[DMM031088C137] Weisz O. A. and Rodriguez-Boulan E. (2009). Apical trafficking in epithelial cells: signals, clusters and motors. *J. Cell Sci.* 122, 4253-4266. 10.1242/jcs.032615 19923269 PMC2779128

[DMM031088C138] Wiegerinck C. L., Janecke A. R., Schneeberger K., Vogel G. F., van Haaften-Visser D. Y., Escher J. C., Adam R., Thoni C. E., Pfaller K., Jordan A. J. et al. (2014). Loss of syntaxin 3 causes variant microvillus inclusion disease. *Gastroenterology* 147, 65-68.e10. 10.1053/j.gastro.2014.04.002 24726755

[DMM031088C139] Yu S., Nie Y., Knowles B., Sakamori R., Stypulkowski E., Patel C., Das S., Douard V., Ferraris R. P., Bonder E. M. et al. (2014). TLR sorting by Rab11 endosomes maintains intestinal epithelial-microbial homeostasis. *EMBO J.* 33, 1882-1895. 10.15252/embj.201487888 25063677 PMC4195784

